# Integrative genome-scale metabolic model of GABAergic neurons reveals metabolic signatures across the mild cognitive impairment – Alzheimer disease continuum

**DOI:** 10.3389/fsysb.2026.1897648

**Published:** 2026-09-03

**Authors:** Andrea Angarita-Rodríguez, Johan H. Largo-González, Julián Pérez-Mejía, Daniel Balcazar, Viviana Vargas-López, Jason A. Papin, Andrés Pinzón, Janneth González

**Affiliations:** 1 Departamento de Nutrición y Bioquímica, Facultad de Ciencias, Pontificia Universidad Javeriana, Bogotá, Colombia; 2 Laboratorio de Bioinformática y Biología de Sistemas, Universidad Nacional de Colombia Bogotá, Bogotá, Colombia; 3 Department of Biomedical Engineering, University of Virginia, Charlottesville, VA, United States; 4 Department of Medicine, Division of Infectious Diseases and International Health, University of Virginia, Charlottesville, VA, United States; 5 Department of Biochemistry & Molecular Genetics, University of Virginia, Charlottesville, VA, United States

**Keywords:** Alzheimer’s disease, GABAergic neurons, genome-scale metabolic models (GEMs), metabolic flexibility, metabolic reprogramming, mild cognitive impairment (MCI), transcriptomic deconvolution

## Abstract

**Introduction:**

Mild cognitive impairment (MCI) represents a prodromal stage of Alzheimer’s disease (AD), but the metabolic mechanisms underlying early neuronal dysfunction remain incompletely understood. GABAergic neurons, which maintain excitatory–inhibitory balance and network stability, exhibit early vulnerability during neurodegeneration, although the metabolic alterations associated with their dysfunction remain poorly characterized.

**Methods:**

We developed a context-specific genome-scale metabolic model (GEM) of human GABAergic neurons across the MCI–AD continuum using deconvolved hippocampal transcriptomic data. By integrating transcriptomic deconvolution with constraint-based modeling, including flux balance analysis (FBA) and flux variability analysis (FVA), we inferred disease-stage-associated metabolic alterations under Control, early MCI (E-MCI), advanced MCI (A-MCI), and AD conditions.

**Results:**

Our analyses suggest progressive remodeling of energy metabolism, the glutamate–glutamine–GABA cycle, redox homeostasis, lipid metabolism, and neuron–astrocyte metabolic interactions. FVA identified reaction-specific changes in feasible flux ranges, indicating remodeling of the feasible metabolic solution space rather than a uniform contraction across pathways. These predicted metabolic alterations were accompanied by transcriptional changes in GABAergic markers and showed qualitative agreement with independent metabolomic observations, supporting their biological plausibility.

**Discussion:**

Overall, this work provides a systems-level computational framework linking transcriptomic alterations with predicted metabolic remodeling in GABAergic neurons and generates experimentally testable hypotheses regarding metabolic dysfunction during progression from MCI to AD.

## 1 Introduction

Mild cognitive impairment (MCI) represents a prodromal stage of Alzheimer’s disease (AD), providing a window in which early pathological processes emerge before irreversible neuronal loss and overt dementia (Ahmed et al., 2008; Etgen et al., 2011; Mufson et al., 2012; Rosenberg et al., 2011). Epidemiological studies estimate an annual conversion rate of approximately 12% from MCI to dementia, highlighting its importance as a target for early diagnosis and therapeutic intervention (Aarsland et al., 2011; Campbell et al., 2013). However, despite advances in biomarker discovery, including cerebrospinal fluid tau and amyloid-β, current approaches remain limited in capturing the system-level alterations that precede disease progression (Chen et al., 2018; Dickerson et al., 2005; Sheinerman et al., 2012).

While the pathogenesis of AD has traditionally been framed in terms of amyloid deposition and tau pathology, emerging evidence suggests that early dysfunction arises at the level of neuronal circuits. In particular, disruption of the excitatory–inhibitory (E/I) balance has emerged as a key feature of early disease stages, highlighting synaptic and network-level dysfunction as early pathogenic events (Gao et al., 2023; Ryan et al., 2021). Within this framework, inhibitory GABAergic neurons play a central role in maintaining circuit stability, synchronization, and cognitive processing.

Consistent with this view, inhibitory neuronal populations, particularly somatostatin-expressing interneurons, have been identified as selectively vulnerable during the early phases of neurodegeneration. Their dysfunction contributes to network hyperexcitability and cognitive decline (Fu et al., 2023; Hoshino et al., 2021; Xu and Wong, 2018).

Importantly, neuronal function is tightly coupled to astrocytic support, particularly through the glutamate–glutamine–GABA cycle and the exchange of metabolic substrates (Gao et al., 2023). Astrocytes regulate neurotransmitter recycling, maintain extracellular homeostasis, and provide metabolic substrates such as lactate to neurons, supporting their high energy demands (Bi et al., 2020; Schousboe et al., 2013). Under pathological conditions such as MCI, astrocytes undergo functional reprogramming partly driven by neuroinflammatory signals, including interleukin-1β (IL-1β), which impair glutamate uptake, alter GABA metabolism, and promote excitotoxicity (De Pittà, 2020; Patel et al., 2019; Valenza et al., 2011). These alterations disrupt the E/I balance and contribute to synaptic dysfunction and neuronal vulnerability.

Despite increasing understanding of circuit-level dysfunction and cell-type-specific vulnerability, a critical gap remains in our understanding of the metabolic mechanisms underlying early GABAergic neuronal dysfunction. This limitation is due, in part, to the inability of experimental methods to directly measure intracellular metabolic fluxes *in vivo*, particularly in specific neuronal subpopulations.

In this context, genome-scale metabolic models (GEMs) provide a systems biology framework for integrating omics data and inferring condition-specific metabolic states (Gu et al., 2019; Terzer et al., 2009). GEMs represent the metabolic network of a cell through stoichiometric reconstructions, enabling the prediction of feasible metabolic flux distributions under defined constraints using approaches such as flux balance analysis (FBA) and flux variability analysis (FVA) (Orth et al., 2010; Sweetlove and George Ratcliffe, 2011). Moreover, the integration of transcriptomic data into GEMs through gene–protein–reaction (GPR) rules allows the contextualization of these models to specific biological conditions, linking transcriptional programs with metabolic phenotype (Filippo et al., 2021).

However, modeling neuronal metabolism in the human brain presents significant technical challenges. Most transcriptomic datasets are derived from bulk brain tissue, which contains a heterogeneous mixture of neuronal, glial and vascular cell populations. This cellular heterogeneity obscures cell-type-specific metabolic signatures, particularly those of vulnerable populations such as GABAergic neurons. Therefore, transcriptomic deconvolution approaches are essential to isolate and reconstruct the metabolic behavior of specific neuronal subtypes from bulk data (Angarita-Rodríguez et al., 2026).

**FIGURE 1:**
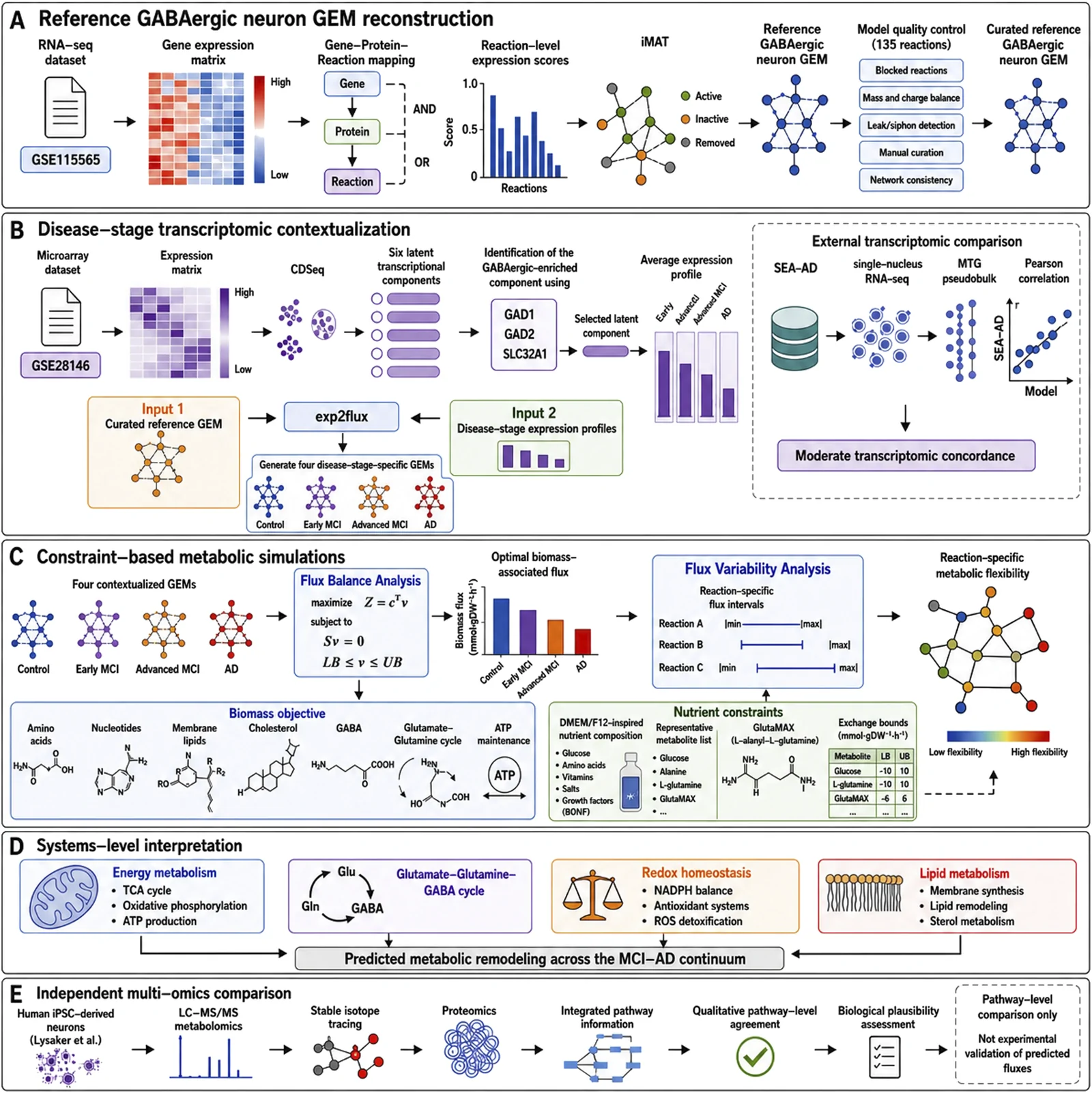
Computational workflow for the reconstruction, contextualization, and systems-level analysis of a human GABAergic neuron genome-scale metabolic model across the MCI–AD continuum. **(A)** Reconstruction of the reference GABAergic neuron genome-scale metabolic model (GEM). RNA-seq data from the human hippocampal dataset GSE115565 were mapped to Recon3D through gene–protein–reaction (GPR) associations to calculate reaction-level expression scores. The iMAT algorithm was used to generate a neuron-specific metabolic network, which was subsequently curated through quality-control procedures including the removal of blocked reactions, mass and charge balance verification, leak/siphon detection, manual curation (135 reactions), and network consistency assessment, yielding the curated reference GABAergic neuron GEM. **(B)** Disease-stage transcriptomic contextualization. Microarray data from the human hippocampal CA1 dataset GSE28146 were deconvoluted using CDSeq to identify latent transcriptional components. The GABAergic-enriched component was identified using the canonical markers *GAD1*, *GAD2*, and *SLC32A1*. Average expression profiles for Control, E-MCI, A-MCI, and Alzheimer’s disease (AD) were integrated with the curated reference GEM using exp2flux to generate four disease-stage-specific metabolic models. External transcriptomic comparison was performed using SEA-AD single-nucleus RNA-seq MTG pseudobulk data to evaluate concordance between predicted and independent transcriptional profiles through Pearson correlation analysis. **(C)** Constraint-based metabolic simulations. Flux Balance Analysis (FBA) was performed using a neuron-specific biomass objective function, whereas Flux Variability Analysis (FVA) was used to estimate reaction-specific flux ranges and metabolic flexibility. Simulations incorporated computational nutrient constraints based on a DMEM/F12-inspired nutrient composition and predefined exchange bounds. **(D)** Systems-level interpretation of simulation results, highlighting alterations in energy metabolism, the glutamate–glutamine–GABA cycle, redox homeostasis, and lipid metabolism associated with progression across the MCI–AD continuum. **(E)** Independent metabolomic comparison. Predicted pathway alterations were qualitatively compared with metabolomic, stable isotope tracing, and proteomic data obtained from human iPSC-derived neurons reported by Lysaker *et al.* to assess biological plausibility at the pathway level. This comparison was intended to evaluate qualitative agreement with independent experimental observations and does not constitute direct experimental validation of predicted metabolic fluxes.

Here, we extend our previously established deconvolution-based metabolic modeling framework to reconstruct a de novo genome-scale metabolic model specific for human GABAergic neurons. While previous metabolic studies of Alzheimer’s disease have primarily relied on bulk-brain reconstructions or generic neuronal models, cell-type-specific metabolic models of vulnerable GABAergic populations remain largely unexplored. By integrating transcriptomic deconvolution with constraint-based modeling, this study provides a systems-level framework to investigate disease-associated metabolic alterations in a neuronal population known to be particularly susceptible to early neurodegenerative processes. This approach enables the identification of GABAergic-specific metabolic signatures and the prediction of metabolic network reprogramming associated with the transition from mild cognitive impairment to Alzheimer’s disease, thereby addressing an important gap in our understanding of cell-type-specific metabolic vulnerability during neurodegeneration (Figure 1).

## 2 Materials and methods

### 2.1 Reconstruction of the GABAergic neuron genome-scale metabolic model

#### 2.1.1 Gene expression mapping to metabolic reactions

To integrate transcriptomic information into the genome-scale metabolic model, RNA-seq data were mapped to metabolic reactions using Gene–Protein–Reaction (GPR) associations derived from the Recon3D model (Brunk et al., 2018). These associations encode the relationships between genes, the proteins they produce, and the reactions they catalyze through Boolean logic, enabling the representation of complex enzymatic mechanisms (Filippo et al., 2021).

Gene expression data were obtained from the publicly available RNA-seq dataset GSE115565, which contains human hippocampal transcriptomic profiles, was used to reconstruct the reference GABAergic neuron genome-scale metabolic model - a different dataset was used to generate disease-stage-specific models or perform comparisons across the MCI–AD continuum as described in Section 2.2.1. Expression values were normalized as Transcripts Per Million (TPM) and log-transformed prior to integration. Genes that could not be matched to Recon3D identifiers during the Entrez-to-Ensembl conversion procedure were excluded from subsequent expression mapping.

Each gene expression value ( $E_{i}$ ) was mapped to its corresponding reactions according to GPR rules using the COBRA Toolbox function *mapExpressionToReactions*. For reactions associated with multiple genes, OR relationships (isoenzymes) were resolved using the maximum expression value, whereas AND relationships (protein complexes) were resolved using the minimum expression value.

Reactions lacking GPR associations could not be directly linked to transcriptomic evidence and therefore did not receive expression scores during the mapping procedure. These reactions were subsequently evaluated during the iMAT optimization process and retained only when necessary to preserve network connectivity, metabolic functionality, and model consistency.

This procedure resulted in a reaction-level expression vector in $R^{n}$ representing the inferred transcriptional support for each metabolic reaction. This reaction-level expression vector served as the input for reconstruction of the reference GABAergic neuron genome-scale metabolic model using the iMAT algorithm.

Two independent transcriptomic datasets were used for different purposes in the study. GSE115565 was exclusively employed for the reconstruction of the reference GABAergic neuron genome-scale metabolic model (GEM) using the iMAT algorithm, because it provides cell-type-specific transcriptomic information suitable for generating a neuronal reference network. In contrast, GSE28146 was used exclusively for disease-stage contextualization because it contains hippocampal CA1 samples spanning Control, E-MCI, A-MCI, and AD conditions and therefore allows modeling of disease progression. Thus, GSE115565 and GSE28146 served complementary but distinct roles within the computational pipeline.

#### 2.1.2 Reconstruction of the reference GABAergic metabolic model using iMAT

A reference GABAergic neuron genome-scale metabolic model was reconstructed using the iMAT algorithm (Zur et al., 2010), as implemented in the COBRA Toolbox v3.0 (Heirendt et al., 2011) through the function *createTissueSpecificModel*. The reaction-level expression vector obtained as described in Section 2.1.1 was used to define activity states within the model.

Reaction expression values were discretized into three categories—high, moderate, and low—based on percentile-derived thresholds from the empirical expression distribution (T_low = 25th percentile; T_high = 75th percentile). This strategy was intended to provide a biologically informed classification without relying on arbitrary cutoffs.

Within the iMAT framework, reactions associated with high expression were preferentially included as active, while those associated with low expression were constrained or penalized. Reactions with intermediate expression were treated as flexible and determined by the optimization process.

This procedure generated a generic tissue-specific genome-scale metabolic model representing human hippocampal GABAergic neurons. The reconstructed network constitutes the reference metabolic framework used throughout this study and was subsequently contextualized using disease-stage-specific transcriptomic profiles to generate models representing Control, early mild cognitive impairment (E-MCI), advanced mild cognitive impairment (A-MCI), and Alzheimer Disease (AD) conditions.

#### 2.1.3 Model consistency, gap-filling, and quality control

To ensure the structural, stoichiometric, and functional consistency of the reconstructed models, a comprehensive quality-control workflow was implemented using functions from the COBRA Toolbox v3.0 (Heirendt et al., 2019).

Blocked reactions were identified using the identifyBlockedRxns function. These reactions, defined as reactions unable to carry flux under any feasible steady-state condition, were used to detect potential network gaps, including missing transport processes and improperly constrained exchange reactions.

Mass and charge balance were evaluated using checkMassChargeBalance, ensuring compliance with conservation laws and reducing the occurrence of biologically implausible flux distributions (Becker and Palsson, 2008). In addition, leak and siphon metabolites were identified using *findMassLeaksAndSiphons*, allowing the detection of metabolites that could be produced or consumed without appropriate network support.

Quality-control analyses identified several infeasible flux cycles associated with ATP-generating and redox-related reactions that required manual inspection and correction. A subset of exchange reactions was found to contribute to ATP leakage artifacts, enabling non-physiological energy generation under closed-system conditions. These reactions were manually curated by revising reaction directionality and refining exchange constraints based on biochemical knowledge and model feasibility requirements.

Exchange reactions were explicitly defined to represent metabolite uptake and secretion between the extracellular environment and cellular compartments. Uptake reactions were constrained according to the defined culture medium and nutrient availability, whereas secretion reactions remained unconstrained unless biological evidence justified additional restrictions.

A total of 135 reactions were manually curated to correct structural inconsistencies in the model, including stoichiometric imbalances, reaction directionality, transport processes, metabolite annotation, and compartmentalization issues. This curation process was particularly important for resolving inconsistencies affecting neuronal metabolism and improving the physiological relevance of the reconstructed network. Detailed information for each curated reaction, including the type of modification performed, biological rationale, and supporting evidence, is provided in Supplementary Table S1. In addition, 263 reactions were retained or further refined during the reconstruction process to ensure network connectivity and support feasible flux distributions under the imposed constraints.

Following model curation, the reconstructed network supported feasible steady-state flux distributions satisfying the mass-balance constraints imposed by the stoichiometric matrix (Sv = 0), indicating that the resulting model was structurally consistent and suitable for subsequent constraint-based analyses (Orth et al., 2010).

To illustrate the biological relevance of the curation process, a subset of representative reactions associated with GABAergic metabolism and transport mechanisms is summarized in Table 1, including pathways involved in glutamate decarboxylation, neurotransmitter transport, glutamate–glutamine cycling, and mitochondrial metabolism.

**TABLE 1:** Curated reactions associated with GABAergic metabolism and transport processes.

Recon3D/BiGG reaction ID	Reaction name	Subsystem	Type	Biological role
GABABGTtc	GABA transport by BGT	Transport	Transport	GABA transport and compartmental redistribution
GABA_import	GABA import	Transport	Transport	Import or redistribution of GABA-related metabolites
GABA_Transaminase	GABA transaminase	GABA metabolism	Metabolic	GABA catabolism and connection with the GABA shunt
GABA_alt_deg	Alternative GABA degradation	GABA metabolism	Metabolic	Alternative route for GABA degradation
EX_4abut [e]	Exchange of 4-aminobutanoate	Exchange	Exchange	Extracellular GABA exchange
EX_glu_L [e]	Exchange of L-glutamate	Exchange	Exchange	Glutamate availability and neurotransmitter cycling
EX_gln_L [e]	Exchange of L-glutamine	Exchange	Exchange	Glutamine availability for glutamate/GABA cycling
GLNB0AT3tc	Glutamine transport by B0AT3	Transport	Transport	Glutamine transport and neurotransmitter precursor availability
OROTGLUt	Orotate–glutamate antiport	Transport	Transport	Glutamate-associated transport and exchange

Representative reactions from the GABAergic neuron-specific metabolic model involved in GABA metabolism, neurotransmitter transport, glutamate–glutamine cycling, and related metabolic processes. Reaction identifiers correspond to the standardized Recon3D/BiGG annotations present in the reconstructed model. For each reaction, the corresponding reaction name, subsystem classification, reaction type, and biological relevance to inhibitory neuronal function are indicated. The complete list of curated reactions and annotations is provided in Supplementary Table S1.

#### 2.1.4 Nutritional input definition

The extracellular constraints were defined using a metabolite composition inspired by Dulbecco’s Modified Eagle Medium (DMEM/F12), supplemented with GlutaMAX, brain-derived neurotrophic factor (BDNF), and fetal bovine serum (FBS), based on previously reported neuronal culture conditions (Bardy et al., 2015; Edwards et al., 2010; Slomp et al., 2023). This formulation was used as a standardized computational framework to support model feasibility and nutrient availability during simulations rather than to reproduce the physiological extracellular milieu of the human brain. Although DMEM/F12-inspired constraints provide a controlled environment for constraint-based modeling, they do not fully recapitulate the complex *in vivo* extracellular conditions present in the human hippocampus or *postmortem* brain tissue. Consequently, model predictions should be interpreted as representing relative metabolic states under a defined nutrient context rather than exact physiological flux distributions. The nutritional formulation was selected to maintain consistency with the previously published reconstruction (Angarita-Rodríguez et al., 2026) and to enable direct comparison between model generations rather than to reproduce the complete biochemical composition of the adult brain extracellular environment.

Rather than representing serum as an undefined component, key nutrients were explicitly mapped to their corresponding metabolite identifiers in the Recon3D model (Brunk et al., 2018), including glucose, amino acids, lipids, and cofactors. Exchange reactions were defined for these metabolites, and their uptake bounds were constrained using values derived from reported medium compositions, thereby enabling a controlled and biologically informed simulation of nutrient availability.

To account for supplementation and variability typically associated with neuronal culture conditions, selected metabolites (e.g., glutamine, branched-chain amino acids, and inositol) were incorporated by extending their allowable uptake ranges within the model. This approach does not represent experimental supplementation *per se*, but rather a computational strategy to explore physiologically plausible metabolic states.

The complete list of metabolites, assigned bounds, and corresponding model identifiers is provided in Supplementary Table S2.

#### 2.1.5 Biomass objective function

The objective function was defined as a neuron-specific biomass maintenance reaction representing the minimal metabolic requirements necessary to sustain cellular viability and functional activity in GABAergic neurons (Feist and Palsson, 2010; Gao et al., 2023; Suyama and Yada, 2018). The biomass formulation was adapted from previously published neuronal biomass reactions and further refined to incorporate metabolic processes characteristic of GABAergic neurotransmission.

Specifically, the biomass reaction included precursors required for amino acid and nucleotide biosynthesis, membrane lipid turnover and cholesterol metabolism, synaptic vesicle maintenance, and cellular redox homeostasis through NADH- and NADPH-dependent processes. In addition, metabolites and reactions associated with the physiological identity of GABAergic neurons were incorporated based on transcriptomic evidence, literature-supported neuronal functions, and pathway-level analyses performed during model contextualization.

To capture key aspects of inhibitory neurotransmission, the biomass formulation included reactions associated with glutamate decarboxylation (GAD1/GAD2-mediated conversion of glutamate to GABA), GABA transport and recycling, and glutamate–glutamine cycling. Energy requirements associated with neurotransmitter synthesis, vesicle recycling, maintenance of membrane potential, and basal cellular homeostasis were represented through ATP consumption terms.

Stoichiometric coefficients were derived from published neuronal biomass formulations and subsequently refined during model reconstruction to ensure compatibility with GABAergic neuronal physiology and network feasibility (Maarleveld et al., 2013). The complete list of metabolites and stoichiometric coefficients included in the biomass reaction is provided in Supplementary Table S3.

Because the objective function can influence flux distributions obtained by flux balance analysis, we performed sensitivity analyses to evaluate the robustness of model predictions to variations in biomass composition. Specifically, all stoichiometric coefficients included in the neuron-specific biomass maintenance reaction were perturbed by ±20% while maintaining all remaining model constraints unchanged. For each perturbed biomass formulation, flux balance analysis (FBA) was repeated for the four contextualized models (Control, E-MCI, A-MCI, and AD), and the resulting objective fluxes together with the principal pathway-level conclusions were compared with those obtained using the original biomass formulation. Major pathway-level conclusions, including alterations in energy metabolism, neurotransmitter-associated pathways, redox homeostasis, and metabolic flexibility, remained qualitatively consistent following perturbation of biomass stoichiometric coefficients, supporting that the reported results were not solely driven by the specific biomass formulation. The sensitivity analysis was designed to evaluate the robustness of the relative metabolic differences among disease stages rather than the absolute magnitude of biomass-associated fluxes.

Additionally, a global reaction-bound sensitivity analysis was performed to evaluate the robustness of the contextualized models to uncertainty in reaction-capacity constraints. Upper and lower bounds of all reactions were uniformly contracted (−20%) and expanded (+20%) while maintaining the biomass formulation, nutrient composition, network topology, and all remaining model constraints unchanged. Flux Balance Analysis was repeated for the four contextualized models, and the resulting objective fluxes were compared across perturbation scenarios. The results of this analysis are presented in Supplementary Figure S1. The purpose of this analysis was to evaluate the robustness of the principal biological conclusions to moderate uncertainty in reaction-capacity constraints rather than to exhaustively quantify all potential sources of modeling uncertainty.

#### 2.1.6 Software environment and computational implementation

All model reconstruction and constraint-based simulations were performed in MATLAB R2025a (MathWorks, Natick, MA, United States of America) using the COBRA Toolbox v3.0 (Heirendt et al., 2019). Linear optimization problems associated with context-specific reconstruction, Flux Balance Analysis (FBA), and Flux Variability Analysis (FVA) were solved using the Gurobi Optimizer v11.0.0 (Gurobi Optimization LLC, Beaverton, OR, United States of America). Default solver tolerances were used unless otherwise specified.

Overall, the methodological framework consisted of three sequential stages: (i) reconstruction of a reference GABAergic neuron genome-scale metabolic model from GSE115565 using the iMAT algorithm, (ii) deconvolution of hippocampal transcriptomic data from GSE28146 to obtain GABAergic-enriched disease-stage-specific transcriptional profiles, and (iii) contextualization of the reference GEM using exp2flux to generate Control, E-MCI, A-MCI, and AD metabolic models across the MCI–AD continuum.

### 2.2 Transcriptomic deconvolution and validation

#### 2.2.1 Deconvolution of bulk transcriptomic data

To obtain a transcriptional component enriched for GABAergic neuronal features, we applied CDSeq (Complete Deconvolution for Sequencing data) (Kang et al., 2021), an unsupervised deconvolution framework that estimates latent cell-type-associated transcriptional components and their relative proportions from bulk transcriptomic measurements without requiring predefined reference signatures.

CDSeq was applied to the publicly available dataset GSE28146 (Blalock et al., 2011), which comprises *postmortem* human hippocampal CA1 samples from control individuals and patients across early and advanced stages of mild cognitive impairment (MCI) and Alzheimer’s disease (AD). All study groups consisted predominantly of older adults, resulting in broadly comparable age distributions across the cohort. Age distributions for each clinical group are shown in Supplementary Figure S2. Gene expression profiles were generated using Affymetrix Human Genome U133 Plus 2.0 microarrays and represented heterogeneous tissue-level measurements containing neuronal and non-neuronal cell populations.

Although CDSeq was originally developed for RNA-seq data, it was adapted here to microarray-derived expression data following preprocessing and normalization procedures. Consequently, the inferred transcriptional components should be interpreted with caution because platform-specific biases associated with microarray measurements may influence deconvolution performance.

Model parameters were optimized to ensure convergence and robust deconvolution (num_cell_types = 6, nu = 0.05, alpha = 5, total_reads = 2 × 10^6^). The selection of six latent transcriptional components was guided by robustness analyses evaluating (i) reconstruction error, (ii) component separability using silhouette coefficients, and (iii) stability of the inferred GABAergic-enriched component across a range of latent component numbers (k = 2–10) (Supplementary Figure S3). Reconstruction error exhibited a marked plateau beyond k = 6, the mean silhouette coefficient reached its maximum at k = 6, and the GABAergic-enriched component remained highly similar across all tested values of k. Collectively, these analyses indicated that k = 6 represents a parsimonious compromise between model fit, component separability, and robustness of the inferred transcriptional signatures. Therefore, six latent components were selected for all subsequent analyses.

The component subsequently selected for metabolic reconstruction was identified based on the enrichment of canonical GABAergic markers, including GAD1, GAD2, and SLC32A1. Therefore, the resulting expression profile should be interpreted as a computationally inferred GABAergic-enriched transcriptional component rather than a purified population of experimentally isolated GABAergic neurons (Banasr et al., 2017). The inferred component likely captures a dominant GABAergic-like transcriptional signal within the bulk tissue while potentially retaining contributions from other cellular sources and deconvolution-related uncertainty.

Because canonical GABAergic markers were used to identify the latent component, downstream interpretation of alterations involving these markers should be considered partially constrained by the component-selection strategy itself. Consequently, the reconstructed metabolic models are intended to represent GABAergic-enriched metabolic states inferred from transcriptomic data rather than direct measurements of metabolism in a pure neuronal population. Accordingly, analyses involving these markers should be interpreted descriptively and not as independent validation of the inferred GABAergic identity.

#### 2.2.2 Identification of the GABAergic neuronal component

Although the underlying model is unsupervised, the identification of the GABAergic neuronal component was guided by prior biological knowledge. Specifically, canonical markers (GAD1, GAD2, and SLC32A1) were used to interpret and select the latent component corresponding to GABAergic neurons (Banasr et al., 2017).

To identify this component, the relative expression of these markers was evaluated across inferred latent profiles (Supplementary Table S4). The component exhibiting the highest expression of GAD1, GAD2, and SLC32A1 was selected as the GABAergic neuronal transcriptional profile.

To evaluate whether this annotation depended exclusively on the canonical markers used for component selection, we performed an independent validation using a panel of 30 inhibitory-neuron markers excluding GAD1, GAD2, and SLC32A1. Marker enrichment was quantified across all six latent CDSeq components within each disease stage using a rank-based Mann–Whitney enrichment analysis with false-discovery-rate correction. Components showing the strongest enrichment for the independent marker signature were compared with the component selected for metabolic reconstruction. This analysis was designed to evaluate the robustness of component annotation and was not intended to compare downstream metabolic reconstructions across all latent components.

#### 2.2.3 Generation of condition-specific transcriptional profiles

To reduce inter-individual variability and obtain representative condition-specific profiles, deconvolved expression matrices were aggregated at the group level. Specifically, gene expression values for the selected GABAergic component were averaged across samples within each clinical group (Control, E-MCI, A-MCI, and AD), resulting in a single representative transcriptional profile per condition.

These group-level profiles were subsequently used for downstream integration into the genome-scale metabolic model.

#### 2.2.4 External qualitative comparison with independent snRNA-seq data (SEA-AD atlas)

To assess the biological consistency of neuronal transcriptional programs inferred through CDSeq, an external qualitative comparison was performed using an independent single-nucleus RNA sequencing (snRNA-seq) dataset from the SEA-AD atlas (Allen Institute for Brain Science) (https://brain-map.org/consortia/sea-ad/our-data), focusing on the middle temporal gyrus (MTG) region.

Given the differences in clinical classification systems across studies, a harmonization strategy based on cognitive severity was implemented. Clinical groups from the GSE28146 dataset were defined according to the original study labels, using Mini-Mental State Examination (MMSE) scores as a reference for disease progression: cognitively normal controls (MMSE ≥25), E-MCI (MMSE ∼20–24), A-MCI (MMSE ∼14–19), and AD (MMSE ≤14).

Using this framework, individuals from the SEA-AD atlas were assigned to equivalent clinical groups based on available metadata, particularly MMSE scores. This process resulted in a final subset of 16 individuals distributed across the four clinical stages. This harmonization approach enabled the alignment of both datasets within a unified disease progression framework, prioritizing biological comparability over strict adherence to original study labels.

Neuronal cells were selected from the SEA-AD dataset (syn26223298) and aggregated into stage-specific pseudobulk profiles using a donor-aware strategy. Gene expression was first averaged at the individual level and subsequently across individuals within each clinical group, minimizing biases related to unequal cell counts per sample.

To ensure comparability between datasets, CDSeq-derived neuronal profiles and snRNA-seq pseudobulk profiles were log-transformed and restricted to a shared set of genes. Expression values were then converted to log fold-change relative to the control condition, allowing the analysis to focus on stage-dependent transcriptional dynamics rather than absolute expression differences. Genes with zero variance across conditions were excluded from the analysis.

#### 2.2.5 Integration of transcriptomic profiles with functional enrichment data

To integrate transcriptomic alterations with functional enrichment results, differentially expressed genes identified across disease stages were linked to significantly enriched Gene Ontology Biological Process (GO-BP) terms obtained from the enrichment analysis (Subramanian et al., 2005). Functional annotations were merged with the corresponding gene expression matrix using a gene-based matching strategy, generating an integrated dataset in which each gene was associated with one or more biological processes.

For each disease condition, gene expression values were aggregated according to their associated GO-BP categories. Mean expression-based scores were subsequently calculated for each biological process, providing condition-specific functional activity profiles. This approach enabled the identification of biological processes displaying coordinated transcriptional alterations across disease progression stages.

**FIGURE 2:**
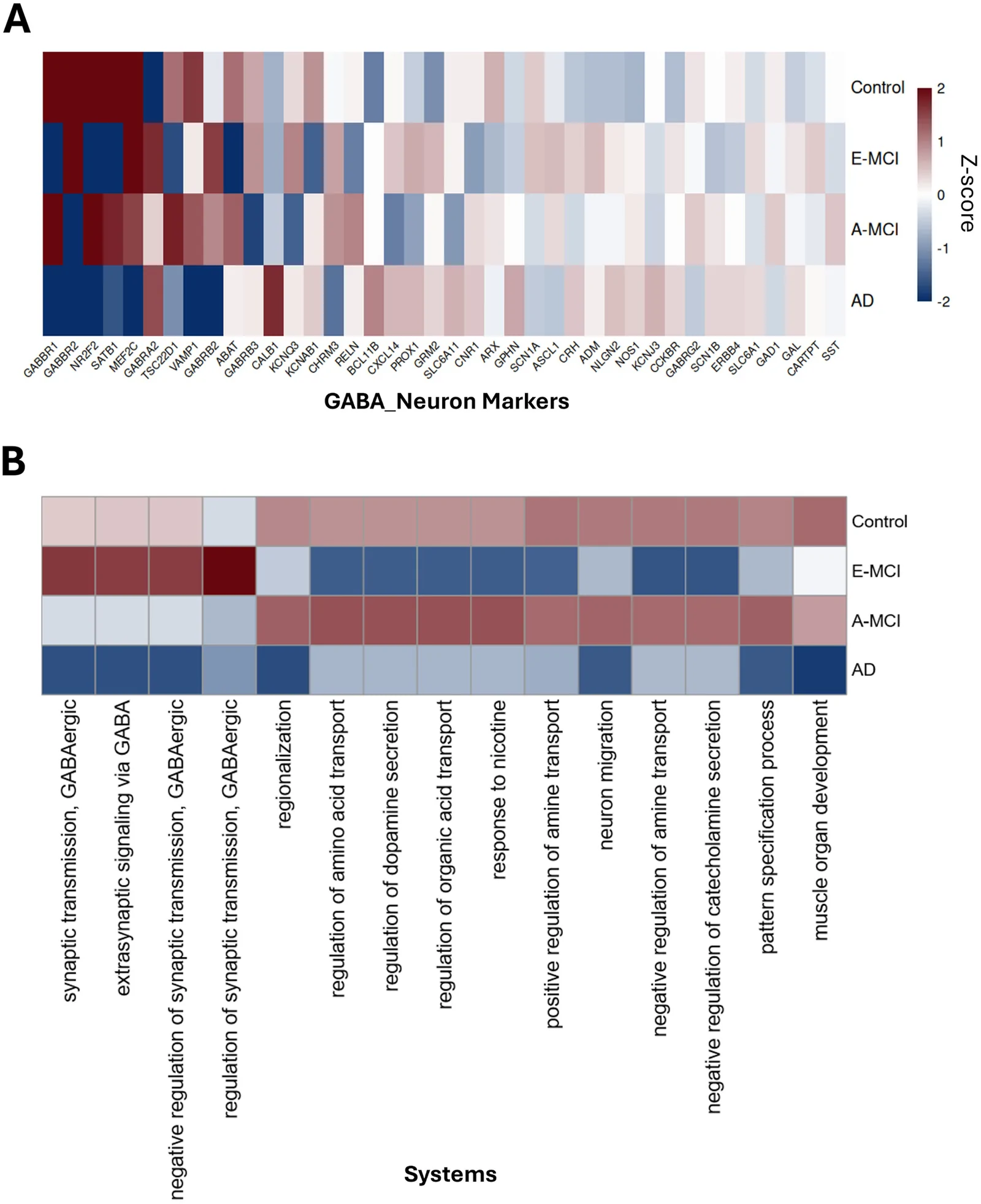
Transcriptomic characterization of the inferred GABAergic neuronal component across the MCI-AD continuum. **(A)** Heatmap showing the normalized expression (Z-score) of GABAergic marker genes across the four clinical conditions (Control, E-MCI, AMCI, and AD). Genes were selected based on established GABAergic neuronal markers and are displayed according to their relative expression across disease stages. **(B)** Functional enrichment analysis of the GABAergic marker gene set. Heatmap displays the normalized enrichment scores (Z-scores) of significantly enriched Gene Ontology biological processes associated with the inferred GABAergic transcriptional component across the four clinical conditions. Functional categories include GABAergic synaptic transmission, neurotransmitter transport, neuronal development, and regulatory processes.

To facilitate comparisons between conditions, expression values were represented as log fold-changes relative to the control group. The resulting matrix was used to generate the visualization presented in Figure 2B, allowing the simultaneous representation of transcriptional changes and their associated functional context throughout the MCI–AD continuum.

### 2.3 Contextualization of the reference GEM across the MCI–AD continuum

#### 2.3.1 Integration of disease-stage transcriptional profiles

The generic GABAergic neuron GEM reconstructed using iMAT served as the reference metabolic network for all downstream analyses. Subsequently, condition-specific transcriptional profiles derived from CDSeq deconvolution of GSE28146 for GABAergic neurons were integrated into this reference model using a modified implementation of the exp2flux algorithm (Osorio et al., 2016). This procedure generated contextualized metabolic models representing Control, E-MCI, A-MCI, and AD conditions.

Importantly, the reconstruction and contextualization procedures were performed sequentially and involved different datasets and algorithms. GSE115565 and iMAT were used exclusively to reconstruct the reference GABAergic neuron GEM, whereas GSE28146, CDSeq deconvolution, and exp2flux were subsequently used to generate disease-stage-specific contextualized models.

#### 2.3.2 Mapping transcriptomic data to metabolic reactions

GABAergic neuron-specific gene expression profiles were integrated into a manually curated genome-scale metabolic model (GEM) of human neurons using a modified implementation of the exp2flux algorithm (Osorio et al., 2016). Transcript abundances, expressed as Transcripts Per Million (TPM), were mapped to metabolic reactions using Gene–Protein–Reaction (GPR) associations derived from the Recon3D model (Brunk et al., 2018).

#### 2.3.3 Definition of transcriptional activity thresholds

To ensure biological relevance, a minimum threshold of TPM >1.0 was applied to define transcriptionally active genes (Lowe et al., 2017). This cutoff is supported by previous studies demonstrating that RNA-seq expression data exhibit a bimodal distribution, separating low-expression transcriptional noise from actively expressed genes.

In particular (Hebenstreit et al., 2011), showed that genes with expression levels below ∼1 TPM are typically associated with background noise, whereas genes above this threshold correspond to biologically meaningful transcriptional activity. Similarly (Wagner et al., 2012), discussed the interpretability of TPM values and highlighted that expression levels around 1 TPM represent the lower bound of reliable gene expression quantification.

Sensitivity analyses using alternative thresholds (TPM >0.5 and TPM >2.0) yielded consistent qualitative results, supporting the robustness of the selected cutoff. Importantly, these transcriptional activity thresholds were used exclusively during exp2flux-based contextualization and are independent from the percentile-based discretization applied during iMAT reconstruction. The percentile thresholds used in iMAT were employed solely to classify reaction activity states during reconstruction of the reference GABAergic GEM, whereas the TPM-based thresholds were used to define transcriptionally active genes during the generation of disease-stage-specific contextualized models. Therefore, the two thresholding schemes serve distinct methodological purposes and are not directly comparable.

#### 2.3.4 Generation of contextualized disease-stage models

Starting from the reference GABAergic GEM, gene expression constraints derived from each disease stage were incorporated through exp2flux to generate four contextualized models corresponding to Control, E-MCI, A-MCI, and AD conditions. These contextualized models constitute the basis for all subsequent Flux Balance Analysis (FBA) and Flux Variability Analysis (FVA) simulations presented in this study. Unless otherwise specified, the term “model” throughout the Results and Discussion sections refers to these contextualized disease-stage models and not to the reference GABAergic reconstruction. Therefore, iMAT was used exclusively to reconstruct the reference GABAergic neuron GEM, whereas exp2flux was used exclusively to generate disease-stage-specific contextualized models. No iMAT optimization was performed during disease-stage contextualization.

### 2.4 Constraint-based simulations

#### 2.4.1 Flux balance analysis (FBA)

To evaluate the functional capacity of each contextualized GABAergic neuron model, Flux Balance Analysis (FBA) was performed under steady-state assumptions ( S·v=0 ), using the neuronal biomass maintenance reaction defined previously as the objective function (Orth et al., 2010).

The biomass formulation was designed to capture key metabolic features of inhibitory neurons, including amino acid and nucleotide turnover, membrane lipid synthesis (notably cholesterol), and redox cofactor requirements (NADH/NADPH).

Importantly, it incorporates GABAergic-specific metabolic demands, such as flux through glutamate decarboxylation (GAD1/GAD2-dependent conversion of glutamate to GABA) and associated glutamate–glutamine cycling. ATP consumption terms were included to account for energy requirements related to synaptic transmission and maintenance of membrane potential.

The complete set of metabolites and their stoichiometric coefficients is provided in Supplementary Table S3.

#### 2.4.2 Flux variability analysis (FVA)

In parallel, Flux Variability Analysis (FVA) was conducted to determine the feasible flux ranges for each reaction under condition-specific constraints (Burgard et al., 2004). This approach enables the characterization of metabolic flexibility while preserving near-optimal functional states.

FVA results were used to assess pathway-level behavior across disease stages, including the identification of alternative route utilization, flux redundancy, and potential bottlenecks. Particular attention was given to pathways relevant to GABAergic neuronal function, such as GABA metabolism, mitochondrial energy production, redox homeostasis, and neurotransmitter cycling.

Because several reactions in Recon3D are associated with multiple enzyme isoforms through Gene–Protein–Reaction (GPR) rules, flux predictions were interpreted at the reaction level rather than as isoform-specific enzymatic activities (Filippo et al., 2021). This consideration is particularly relevant for the lactate dehydrogenase reaction (LDH_L), which is associated with multiple lactate dehydrogenase isoforms, including LDHA and LDHB, and catalyzes the reversible interconversion of lactate and pyruvate (Equation 1):

$$\text{lactate}+{\text{NAD}}^{+}\;⇌\text{ pyruvate}+\text{NADH}+H^{+}$$ (1)

Consequently, flux predictions associated with LDH_L were interpreted as representing the feasible metabolic capacity for lactate–pyruvate interconversion rather than directional changes in lactate production or consumption.

### 2.5 Qualitative plausibility assessment using independent metabolomic observations

To assess the biological plausibility of the inferred metabolic states, model-derived predictions were compared with independent metabolomic observations reported by (Lysaker et al., 2025). This study combined stable isotope tracing, metabolomics, and proteomics analyses in human induced pluripotent stem cell (iPSC)-derived neuronal and astrocytic models carrying the APOE4 genotype, providing experimental evidence of alterations in energy metabolism, mitochondrial function, neurotransmitter-associated pathways, and redox-related processes.

Because the reference study reported metabolite abundances, isotope-labeling patterns, and pathway-level alterations rather than intracellular metabolic fluxes, plausibility assessment was performed qualitatively at the pathway level rather than through direct quantitative comparisons. Consequently, model predictions were compared according to their consistency with experimentally reported alterations in biological processes associated with central carbon metabolism, tricarboxylic acid cycle activity, glutamate metabolism, aspartate-associated metabolism, malate–aspartate shuttle function, and NAD-related pathways.

Flux Variability Analysis (FVA) results were used to characterize the feasible metabolic solution space associated with each contextualized model (Burgard et al., 2004). Pathways exhibiting reaction-specific alterations in flux variability across disease stages were compared with experimentally observed metabolic alterations reported in the reference study. Qualitative concordance was defined as broad agreement in the direction of pathway-level alterations between model-derived metabolic remodeling and independently reported experimental observations. Because the experimental study measured metabolite abundances and isotope-labeling patterns rather than intracellular fluxes, these comparisons were interpreted exclusively as qualitative plausibility assessments and not as direct experimental validation of predicted metabolic fluxes.

**FIGURE 3:**
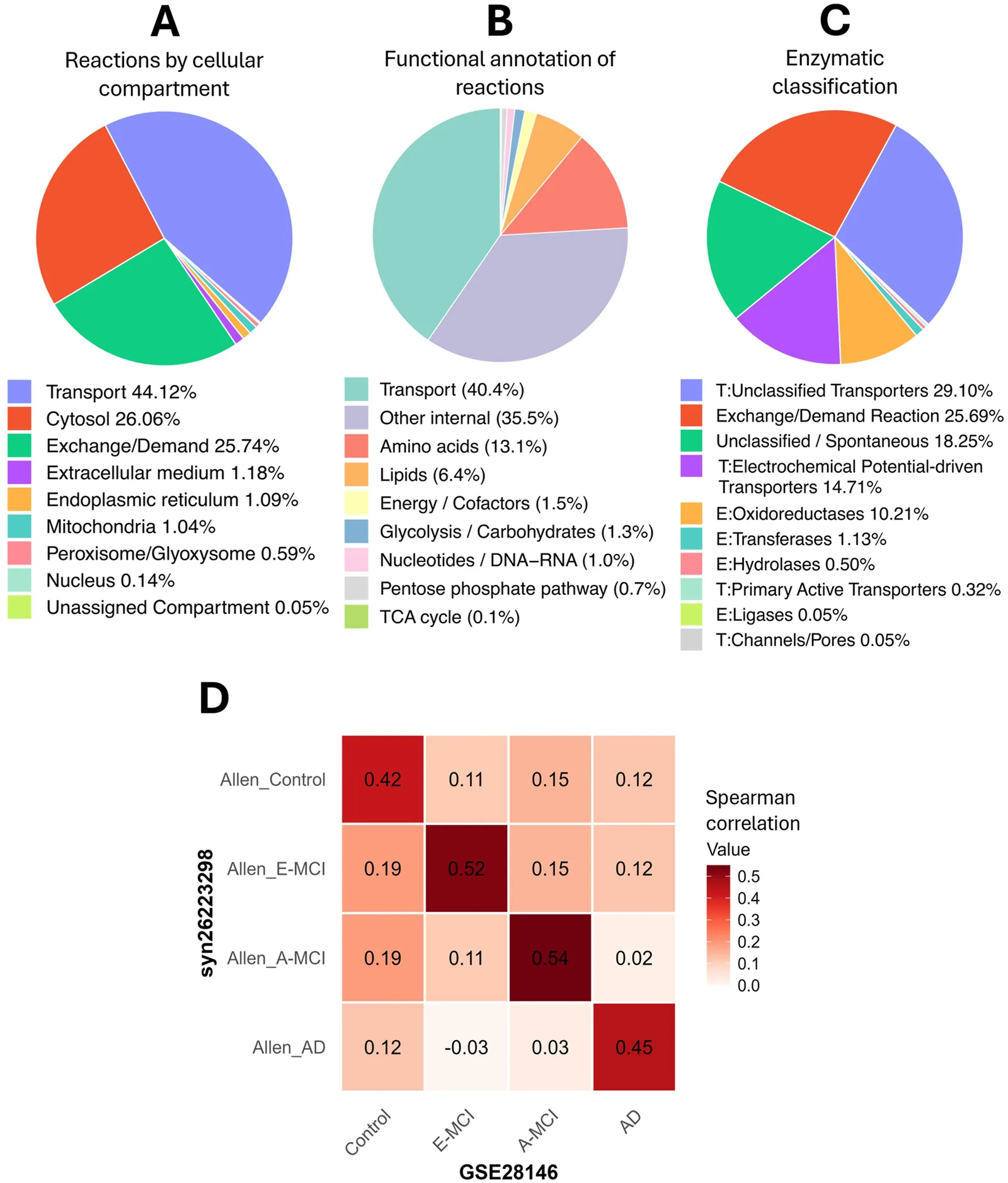
Structural characterization of the contextualized GABAergic genome-scale metabolic model and external validation of the inferred GABAergic transcriptional component. **(A)** Distribution of reactions in the contextualized GABAergic GEM according to their assigned cellular compartment. Percentages indicate the proportion of reactions associated with each compartment. **(B)** Functional classification of reactions into major metabolic categories according to subsystem annotation. Pie chart segments represent the proportion of reactions assigned to each metabolic category. **(C)** Classification of reactions according to enzyme or transporter. Transport, exchange/demand, spontaneous reactions, and enzyme classes are shown as percentages of the total reactions retained in the contextualized model. **(D)** External validation of the inferred GABAergic-enriched transcriptional component using independent single-nucleus RNA sequencing (snRNA-seq) data from the SEA-AD cohort. Heatmap shows Spearman correlation coefficients between the deconvolved GABAergic transcriptional component derived from GSE28146 and the corresponding clinical-stage-specific GABAergic populations from the Allen Institute SEA-AD dataset. Higher correlation values along the diagonal indicate greater concordance between matching disease stages.

To further support the biological identity of the inferred GABAergic component beyond the initial marker-based selection (Banasr et al., 2017), we performed an independent external validation using inhibitory neuronal populations from the SEA-AD single-nucleus RNA-seq atlas. Canonical GABAergic marker genes were evaluated in the corresponding inhibitory neuronal populations, confirming that the inferred transcriptional component retained the expected inhibitory neuronal identity (Supplementary Figure S4). This analysis served exclusively to validate the cellular identity of the inferred GABAergic component and was performed independently of the disease-stage validation presented in Figure 3D, we performed an independent external validation using inhibitory neuronal populations from the SEA-AD single-nucleus RNA-seq atlas. Canonical GABAergic marker genes were evaluated in the corresponding inhibitory neuronal populations, confirming that the inferred transcriptional component retained the expected inhibitory neuronal identity (Supplementary Figure S4). This analysis served exclusively to validate the cellular identity of the inferred GABAergic component and was performed independently of the disease-stage validation presented in Figure 3D.

## 3 Results

### 3.1 Structural and functional characterization of the GABAergic neuron-specific metabolic model

Following reconstruction with iMAT, a reference GABAergic neuron genome-scale metabolic model comprising 1,548 metabolites, 2,203 reactions, and 3,290 unique genes was obtained. This reference network subsequently served as the scaffold for generating the contextualized Control, E-MCI, A-MCI, and AD models through integration of disease-stage-specific transcriptional profiles. Unless otherwise indicated, the structural characteristics described in this section refer to the reference GABAergic reconstruction, whereas metabolic simulations and comparisons across disease stages were performed using the contextualized models.

The distribution of reactions across cellular compartments (Figure 3A) revealed that the cytosol emerged as a central hub of metabolic integration because it contains the convergence of amino acid metabolism, glycolysis, neurotransmitter biosynthesis, and redox pathways. In particular, glutamine, glutamate, and GABA metabolism converge within the cytosolic compartment before redistribution toward mitochondrial metabolism or extracellular exchange, underscoring the central role of cytosolic processes in sustaining inhibitory neurotransmission and metabolic homeostasis. In contrast, specialized organelles such as mitochondria (1.04%), endoplasmic reticulum (1.09%), and peroxisome/glyoxysome (0.59%) were less represented, while the nucleus (0.14%) and unassigned compartments (0.05%) contributed minimally. This pattern suggests a functional organization oriented towards transport and metabolic integration.

Functional annotation of reactions (Figure 3B) further supported this organization, with transport processes accounting for 40.4% of reactions. Detailed examination of these reactions revealed that transport functions were predominantly associated with amino acid exchange, monocarboxylate transport, and mitochondrial carrier systems (Supplementary Figure S5; Supplementary Table S5). Amino acid transport reactions included glutamine, glycine, serine, alanine, branched-chain amino acids, and aromatic amino acids, whereas monocarboxylate transport reactions involved lactate, pyruvate, and ketone bodies. In addition, numerous mitochondrial carrier reactions mediated the exchange of tricarboxylic acid intermediates, including citrate, α-ketoglutarate, succinate, and malate. Together, these findings indicate that the reconstructed GABAergic network is strongly dependent on extracellular metabolite exchange and compartmentalized metabolite trafficking required for neurotransmitter synthesis, energy metabolism, and neuron–astrocyte metabolic interactions.

Enzymatic classification (Figure 3C) revealed the dominance of transport and exchange processes. Transport reactions represented approximately 44% of the total, including unclassified transporters (29.10%) and electrochemical gradient-driven transporters (14.71%), with smaller contributions from primary active transporters and channels. Exchange and demand reactions constituted 25.69%, indicating a strong reliance on metabolite exchange with the extracellular environment.

Additionally, 18.25% of reactions corresponded to unclassified or spontaneous processes, reflecting limitations in annotation and potential gaps in metabolic knowledge. Classical enzymatic classes were comparatively underrepresented, with oxidoreductases (10.21%) being the most prominent, followed by transferases (1.13%), hydrolases (0.50%), and ligases (0.05%). Overall, transport and exchange reactions represented the predominant functional categories within the reconstructed network, highlighting potential metabolic vulnerabilities under pathological conditions.

### 3.2 Transcriptomic deconvolution and qualitative assessment of the GABAergic neuronal component

Transcriptomic deconvolution was performed as described in Section 2.2.1 to infer GABAergic-enriched transcriptional components from the GSE28146 dataset. Robustness analyses supported the selection of six latent components (Supplementary Figure S3). Before evaluating disease-stage concordance, we first confirmed the biological identity of the inferred transcriptional component. In addition to the canonical GABAergic markers used during component selection, we performed an independent validation using a panel of 30 inhibitory-neuron markers that excluded GAD1, GAD2, and SLC32A1 (Supplementary Figures S4 and S6; Supplementary Table S6). The independently selected marker signature confirmed the strongest enrichment of the selected component in E-MCI and AD, while the selected component remained significantly enriched in A-MCI. In the Control stage, the independent signature identified a closely related latent component, indicating greater overlap among neuronal transcriptional components under physiological conditions. Collectively, these results support that the GABAergic annotation was not driven exclusively by the three canonical marker genes used during component identification.

A qualitative comparison of the inferred GABAergic-enriched transcriptional component with independent single-nucleus RNA sequencing (snRNA-seq) data from the SEA-AD atlas (Allen Institute for Brain Science) demonstrated moderate concordance across disease stages (Figure 3D). Higher correlations were observed between matched clinical conditions, including Control–Control (r = 0.42), E-MCI–E-MCI (r = 0.52), A-MCI–A-MCI (r = 0.54), and AD–AD (r = 0.45). In contrast, non-equivalent comparisons exhibited lower correlations. These findings suggest partial preservation of disease-associated neuronal transcriptional trends across independent datasets and provide an external plausibility check of the inferred transcriptional component. The moderate correlations observed across datasets likely reflect both shared disease-associated transcriptional programs and context-dependent differences arising from distinct brain region (hippocampal CA1 versus middle temporal gyrus), experimental platform (bulk microarray versus single-nucleus RNA sequencing), cellular resolution, and cohort composition. Consequently, these comparisons were intended to evaluate preservation of disease-stage-associated transcriptional patterns rather than transcriptome-wide equivalence between datasets.

To further strengthen the external validation, three complementary analyses were performed. First, we quantified stage-matched correlation coefficients between the CDSeq-derived GABAergic transcriptional component and the corresponding SEA-AD pseudobulk profiles. Second, bootstrap-derived 95% confidence intervals were estimated for these correlation coefficients (Supplementary Figure S6), providing an assessment of their robustness despite the limited number of harmonized individuals. Third, the disease-stage correlation analysis was complemented by an independent validation of the biological identity of the inferred GABAergic component using canonical GABAergic marker genes within inhibitory neuronal populations from the SEA-AD dataset. Together, these analyses support both the preservation of disease-associated transcriptional dynamics across datasets and the biological identity of the inferred GABAergic transcriptional component.

A gradual similarity pattern was observed across clinical stages, with intermediate conditions (E-MCI and A-MCI) displaying partial correlations with multiple groups. This pattern is consistent with progressive transcriptional transitions and suggests that the inferred GABAergic neuronal component captures continuous changes across disease stages. The moderate correlation values may also be partially explained by regional specificity, as the validation dataset corresponds to the middle temporal gyrus (MTG), whereas the deconvolution analysis was performed using hippocampal CA1 samples.

Consistent with these observations, analysis of GABAergic neuronal markers (Figure 2A) revealed stage-dependent transcriptional reorganization. Functional enrichment analysis (Figure 2B) further demonstrated alterations in key neuronal processes, including energy metabolism, metabolite transport, and neurotransmission, with progressive disruption in advanced stages such as AD (Angarita-Rodríguez et al., 2026).

### 3.3 Transcriptomic dynamics and functional alterations across disease progression

Progressive alterations in GABAergic markers were observed across disease stages (Figure 2A), particularly affecting genes encoding ionotropic and metabotropic receptors (GABRA2, GABRB2, GABRB3, GABRG2, GABBR1, and GABBR2). To quantify these changes, gene-level variability and monotonic trends were assessed using standard deviation (SD) and Spearman correlation (ρ), respectively. SD was used as a descriptive measure of the magnitude of expression changes across the four ordered disease stages, whereas Spearman correlation was used to characterize increasing or decreasing monotonic trajectories. Because the GABAergic-enriched component was identified using the canonical markers GAD1, GAD2, and SLC32A1, expression patterns involving these markers and closely related genes should be interpreted with caution, as the component-selection strategy may partially influence the observed transcriptional trends. Consequently, the results presented in this section should be interpreted as a descriptive characterization of the inferred GABAergic-enriched transcriptional profile rather than as independent evidence of disease-associated changes in these marker genes.

This analysis identified several highly dynamic genes across the disease continuum, including GABBR1 (SD = 2.31; ρ = −0.45), GABBR2 (SD = 2.31; ρ = −0.89), NR2F2 (SD = 2.31; ρ = −0.45), SATB1 (SD = 2.13; ρ = −0.40), and MEF2C (SD = 1.93; ρ = −1.00), indicating substantial expression variability across the disease continuum. Conversely, genes such as NOS1 (ρ = 1.00), CALB1 (ρ = 0.80), BCL11B (ρ = 0.80), CNR1 (ρ = 0.80), GPHN (ρ = 0.80), NLGN2 (ρ = 0.80), and CRH (ρ = 0.80) displayed positive monotonic trajectories across the ordered disease stages.

In contrast, several genes associated with inhibitory neurotransmission and synaptic function, including MEF2C (ρ = −1.00), GABBR2 (ρ = −0.89), SCN1A (ρ = −0.80), VAMP1 (ρ = −0.80), and GABBR1 (ρ = −0.45), displayed negative monotonic trajectories across the ordered disease stages. Together, these patterns suggest progressive alterations in transcriptional programs related to inhibitory signaling and synaptic maintenance. Complete results, including standard deviation and Spearman correlation coefficients for all genes, are provided in Supplementary Tables S7 and S8.

The global organization of the heatmap further revealed a stage-dependent transcriptional gradient across disease progression (Figure 2A). Control samples displayed a comparatively preserved expression profile characterized by higher expression of genes associated with inhibitory neurotransmission and synaptic maintenance, whereas AD samples exhibited a broader pattern of transcriptional repression affecting multiple components of GABAergic signaling. Intermediate conditions (E-MCI and A-MCI) showed partial and heterogeneous alterations, frequently displaying intermediate expression states between control and AD. This progressive transition was particularly evident for genes involved in receptor signaling, synaptic vesicle dynamics, and neuronal identity, supporting the presence of gradual transcriptional reprogramming rather than an abrupt stage-specific disruption. Because both SD and Spearman correlation analyses were computed using only four ordered clinical conditions, these metrics should be interpreted exclusively as descriptive assessments of transcriptomic variability and monotonic trends across disease progression rather than as formal statistical inference or evidence of statistically significant associations. Accordingly, values of |ρ| = 1.00 indicate perfect monotonic ordering across the four disease stages but should not be interpreted as evidence of statistical significance. Therefore, the transcriptomic patterns described in this section are intended to characterize the direction and consistency of gene expression changes across disease progression rather than to establish statistically significant associations.

### 3.4 Functional alterations in GABAergic-related processes across disease progression

Functional enrichment analysis revealed marked and stage-dependent alterations in biological processes associated with GABAergic neuronal activity and neurotransmission (Figure 2B). The overall organization of the enrichment heatmap revealed coordinated clustering of biological processes associated with neurotransmission, metabolite transport, synaptic regulation, and neuronal signaling across disease stages. Control samples exhibited comparatively preserved enrichment patterns, whereas AD samples showed a broader reduction in pathway-associated activity scores across multiple functional categories. Intermediate conditions (E-MCI and A-MCI) displayed partially preserved profiles with heterogeneous transitions between control and AD states, supporting the presence of progressive functional reprogramming rather than abrupt pathway disruption.

Processes directly associated with GABAergic synaptic transmission showed distinct modulation across disease stages. In early condition, several pathways, including synaptic transmission via GABAergic signaling and extra synaptic signaling, displayed relatively higher activity levels as compared to advanced stages. However, a progressive reduction in these processes was observed as the disease advanced toward MCI and AD, consistent with altered inhibitory synaptic signaling across disease stages.

Similarly, pathways related to the regulation of synaptic transmission exhibited a clear shift across conditions. Both positive and negative regulatory processes showed differential modulation, with a tendency toward decreased activity in later stages, particularly in AD. This pattern was consistent across multiple regulatory layers, suggesting a broad alteration in synaptic control mechanisms.

Processes associated with neurotransmitter transport and secretion, including the regulation of dopamine secretion, organic acid transport, and amine transport, also demonstrated progressive changes. These pathways showed relatively preserved or moderately elevated activity in control and early stages, followed by a gradual decline in MCI and a more pronounced reduction in AD. This trend was consistent across both general and specific transport-related functions, indicating a disruption in neurotransmitter handling and cellular exchange mechanisms.

In addition, pathways related to the regulation of amine transport and catecholamine secretion exhibited a similar trajectory. Both positive and negative regulatory processes showed decreasing enrichment scores along disease progression, suggesting alterations in the fine-tuning of neurotransmitter dynamics. Processes linked to neuronal migration and pattern specification displayed more variable behavior, with intermediate stages (E-MCI and A-MCI) often showing partial retention of activity compared to controls, followed by a marked reduction in AD. This pattern indicates stage-specific modulation rather than a strictly monotonic trend.

### 3.5 Impact of contextualization on neuronal metabolic fluxes

A progressive reduction in optimal biomass-associated flux values was observed from control to pathological conditions (Table 2). After regenerating the contextualized models using reaction-specific expression-derived constraints, flux balance analysis (FBA) showed the highest biomass objective value in Control (0.100 mMgDW−1 h−1), followed by E-MCI (0.086), A-MCI (0.043), and AD (0.039). This corrected analysis supports a decline in GABAergic neuronal metabolic capacity, with the most pronounced reduction occurring between early and advanced MCI stages, consistent with progressive alterations in biosynthetic and energetic functions during disease progression.

**TABLE 2:** Progressive reduction in maintenance-associated biomass flux across disease stages and sensitivity to biomass formulation in the GABAergic neuron-specific model.

Biomass perturbation	Control	E-MCI	A-MCI	AD
−20%	0.125	0.108	0.0538	0.0488
Baseline	0.100	0.086	0.0430	0.0390
20%	0.0833	0.0717	0.0358	0.0325

Flux Balance Analysis (FBA) predicted a progressive reduction in the neuron-specific biomass objective across the MCI–AD continuum, with the highest flux observed in Control, followed by E-MCI, A-MCI, and AD. To evaluate the robustness of these predictions, all stoichiometric coefficients of the neuron-specific biomass reaction were simultaneously perturbed by ±20%, and FBA was repeated under identical constraints. Although absolute objective flux values varied proportionally with the imposed perturbations, the relative ordering of clinical conditions (Control > E-MCI > A-MCI > AD) remained unchanged across all scenarios. These results indicate that the inferred decline in maintenance-associated metabolic capacity is robust to reasonable variations in the biomass formulation and is not driven by a specific parameterization of the objective function.

Sensitivity analysis of the biomass objective demonstrated that perturbation of all stoichiometric coefficients in the neuron-specific biomass reaction by ±20% produced only limited quantitative variation in the optimal objective fluxes while preserving the relative ordering of the contextualized models (Control > E-MCI > A-MCI > AD) under all perturbation scenarios. Likewise, the principal pathway-level interpretations derived from FBA and FVA remained unchanged, indicating that the predicted reductions in maintenance-associated metabolic capacity and reaction-specific metabolic remodeling were robust to reasonable variations in biomass composition.

To further evaluate the robustness of the contextualized metabolic models, we performed a complementary global sensitivity analysis by uniformly contracting (−20%) and expanding (+20%) the upper and lower bounds of all reactions while preserving the biomass formulation and all remaining model constraints. As shown in Supplementary Figure S1, the expected quantitative variation in the optimal biomass-associated fluxes was observed under both perturbation scenarios. Importantly, the relative ordering of disease stages (Control > E-MCI > A-MCI > AD) remained unchanged across all simulations, indicating that the principal metabolic conclusions are robust not only to biomass formulation but also to moderate uncertainty in reaction-capacity constraints.

Reduced optimal flux values should be interpreted as computationally constrained feasible states derived from condition-specific model contextualization rather than direct measurements of cellular metabolic activity.

Flux balance analysis (FBA) revealed lower flux values in several metabolic reactions associated with energy metabolism and mitochondrial function, including ATP synthase, pyruvate kinase, hexokinase, lactate dehydrogenase, and malate dehydrogenase (Table 3). In the case of lactate dehydrogenase, the LDH_L reaction represents the reversible interconversion of lactate and pyruvate and is associated with multiple lactate dehydrogenase isoforms within Recon3D. Therefore, the observed reductions should be interpreted as alterations in lactate-associated metabolic capacity rather than as direct evidence of reduced lactate production or consumption. Alterations were also observed in reactions associated with glutamine, glutamate, and GABA metabolism, as well as pathways related to glutathione, cholesterol, and glycerophosphocholine metabolism.

**TABLE 3:** Flux balance analysis (FBA) reveals coordinated metabolic alterations in GABAergic neuron models across disease stages.

Metabolite/Process	RxnID	FBA Reaction	Control	E-MCI	A-MCI	AD	Interpretation
ATP/Bioenergetics	r1116	ATP exporter (ATP-E)	0.006	0.363	0.178	0.007	Altered ATP handling and extracellular ATP signaling during disease progression
Glucose	HEX1	Hexokinase (D-Glucose:ATP)	0.166	0.276	0.275	0.023	Non-linear remodeling of glucose utilization with a marked reduction in AD.
Lactate	LACLt	Lactate transport	0.566	0.129	0.011	0.566	Altered lactate transport suggests disruption of the astrocyte–neuron lactate shuttle
Malate/TCA cycle	MDH	Malate dehydrogenase	0.412	0.360	0.188	0.006	Progressive reduction in mitochondrial metabolic flexibility culminating in AD.
Glutamine	EX_gln_L [e]	Exchange L-Glutamine	0.453	0.496	2.657	0.085	Major perturbation of the glutamate–glutamine cycle with reduced flexibility in AD.
Glutamate	OROTGLUt	Orotate–Glutamate antiport	0.009	0.557	0.564	0.031	Increased glutamate-associated transport in intermediate stages followed by reduction in AD.
GABA	GABABGTtc	GABA transport by Bgt	0.012	0.006	0.027	0.006	Stage-specific remodeling of inhibitory neurotransmission
Glutathione	RE2296C	Glutathione transferase	0.021	0.113	0.006	0.006	Altered redox metabolism with highest flexibility in E-MCI.
Cholesterol	CHSTEROLtrc	Cholesterol transport	0.088	0.566	0.148	0.031	Strong lipid metabolic remodeling in E-MCI followed by reduced transport in AD.
Glycerophosphocholine	VANILLACc	Formation of Vanil-lactate	0.085	0.272	0.150	0.018	Altered aromatic metabolite remodeling with reduced capacity in AD.

Reaction-level flux analysis shows a consistent reduction in key bioenergetic, mitochondrial, and neurotransmission-related processes from control to AD. Decreased fluxes in ATP synthesis, glycolysis (hexokinase), TCA cycle (malate dehydrogenase), and lactate metabolism indicate impaired energy production and mitochondrial dysfunction. Concurrent reductions in glutamine, glutamate, and GABA-associated fluxes suggest disruption of the glutamate–glutamine cycle and inhibitory neurotransmission. Additionally, alterations in glutathione, cholesterol, and glycerophosphocholine metabolism reflect compromised redox homeostasis and lipid remodeling. Together, these results support a progressive metabolic impairment linking bioenergetic failure, oxidative stress, and neurotransmitter imbalance in neurodegeneration.

**TABLE 4:** Qualitative plausibility assessment of inferred metabolic remodeling using independent metabolomic observations.

Metabolic module	Independent observations (Lysaker et al., 2025)	GEM reaction proxy	Control FVA amplitude	AD FVA amplitude	Δ% vs. control	Biological interpretation	Qualitative concordance
Glutamine/Glutamate metabolism	Increased glutamate labeling in APOE4 neurons and astrocytes	EX_glu_L [e], EX_gln_L [e]	7.0	2.42	−65%	Reduced feasible flux space associated with glutamate–glutamine cycling	Partial
Lactate-associated metabolism	Altered lactate utilization and mitochondrial dysfunction	LACr	6.80	2.00	−71%	Reduced flexibility in lactate–pyruvate interconversion	Partial
Ammonium metabolism	Altered nitrogen handling associated with mitochondrial dysfunction	EX_nh4 [e]	5.80	1.70	−71%	Reduced flexibility in nitrogen metabolism and amino acid turnover	Partial
Chloride-associated transport	Altered ion homeostasis in neurodegeneration	CLFORTe	2.00	0.25	−88%	Marked reduction in transport flexibility associated with ionic balance	Partial
GABA transport	Altered inhibitory neurotransmission and GABAergic dysfunction	GABABGTtc	0.85	0.20	−76%	Reduced feasible space for GABA transport and recycling	Partial
Glutamine transport	Impaired neurotransmitter precursor availability	GLNBOAT3tc	0.70	0.15	−79%	Reduced glutamine transport capacity potentially affecting neurotransmitter cycling	Partial
Pyruvate transport	Altered mitochondrial metabolism and substrate utilization	PYRSMCT1	0.55	0.12	−78%	Reduced transport flexibility associated with energy metabolism	Partial

Model-derived alterations in flux variability were qualitatively compared with independent metabolomic observations reported by Lysaker et al. (2025). Because the experimental study quantified metabolite abundances and isotope-labeling patterns rather than intracellular metabolic fluxes, concordance should be interpreted as pathway-level consistency and biological plausibility rather than direct experimental validation of predicted flux states.

Together, these results indicate substantial differences in feasible flux distributions across disease stages within the contextualized models.

### 3.6 Progressive metabolic disruption in GABAergic neurons

**FIGURE 4:**
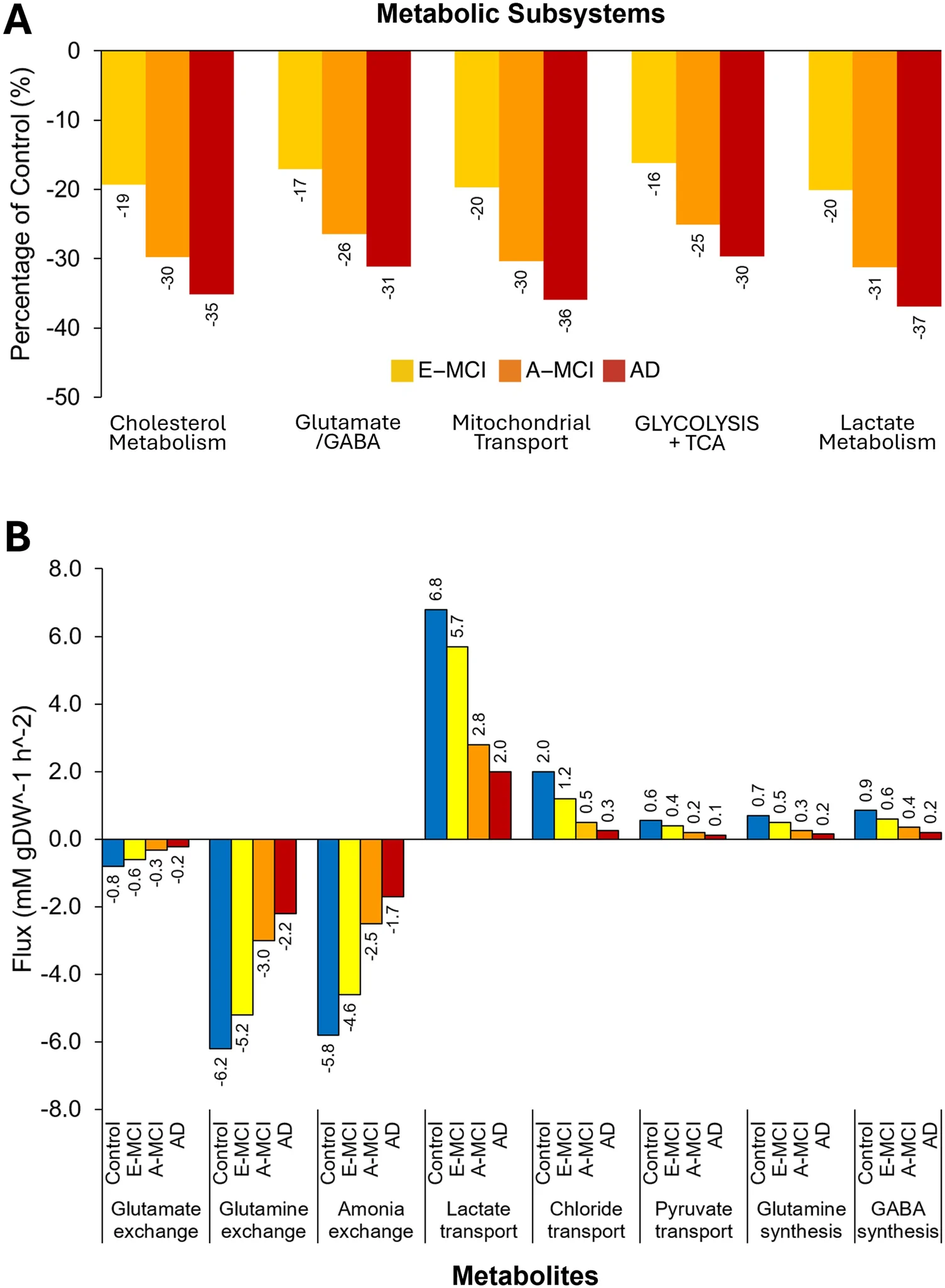
Changes in representative metabolic subsystems and flux variability across the MCI–AD continuum. **(A)** Relative changes in representative metabolic subsystems in the contextualized GABAergic genome-scale metabolic model. Bars indicate the percentage reduction with respect to the control model for cholesterol metabolism, glutamate/GABA metabolism, mitochondrial transport, glycolysis/TCA cycle, and lactate metabolism across E-MCI, A-MCI, and AD. **(B)** Flux variability analysis (FVA) of representative reactions associated with GABA transport, glutamate metabolism, glutamine exchange, lactate transport, α-ketoglutarate exchange and antiport, NAD metabolism, pentose phosphate pathway (G6PDH and GND), and glutathione transferase (GST). Bars represent the minimum and maximum feasible fluxes (mmol gDW^-1^ h^-1^) predicted for each clinical stage under identical model constraints. Progressive changes in the feasible flux ranges illustrate alterations in the solution space predicted by the contextualized GABAergic GEM across disease progression.

Metabolic pathway analysis (Figure 4A) revealed a reaction-specific and stage-dependent metabolic remodeling across pathways associated with energy metabolism, neurotransmitter cycling, and redox homeostasis. The heatmap showed a progressive transition from control to AD conditions, with intermediate stages (E-MCI and A-MCI) displaying partially preserved metabolic profiles. Pathways associated with glycolysis, neurotransmitter metabolism, redox homeostasis, and transport processes exhibited heterogeneous and reaction-specific alterations across disease stages.

This pattern reflects progressive metabolic remodeling characterized by alterations in energy metabolism, mitochondrial-associated pathways, and neurotransmitter cycling.

These metabolic alterations are consistent with the transcriptional reprogramming observed in GABAergic-related genes. The progressive downregulation of GABBR1 and GABBR2 aligns with reduced glutamate/GABA metabolism, while decreased expression of transcriptional regulators such as MEF2C and NR2F2 parallels impairment of energy-related pathways.

Conversely, upregulation of genes such as NOS1 and NLGN2 may reflect compensatory transcriptional responses associated with altered metabolic states.

### 3.7 Flux variability and metabolic flexibility

Flux variability analysis (FVA) was recalculated using the corrected reaction-specific contextualized models generated from expression-derived reaction-specific constraints. In contrast to the previous analysis, the updated results no longer supported a uniform contraction of feasible flux ranges across unrelated pathways. Instead, the corrected models revealed reaction-specific and stage-dependent alterations in metabolic flexibility across the MCI–AD continuum (Figure 4B).

To avoid ambiguity, FVA intervals are reported as minimum flux, maximum flux, and flux range (FVAmax − FVAmin), because several reactions exhibited asymmetric feasible flux distributions. The updated analysis demonstrated heterogeneous patterns of metabolic remodeling, with some reactions remaining relatively stable across conditions, whereas others displayed marked stage-dependent changes in their feasible flux intervals.

Pathways associated with glucose metabolism, glutamine exchange, GABA transport, lipid metabolism, and redox-associated processes exhibited the most evident alterations in flux variability. For example, glucose-associated reactions showed pronounced condition-specific changes, whereas cholesterol transport and glutathione-associated reactions exhibited increased flexibility in advanced disease stages. In contrast, other reactions, including glutamate exchange and glycerophosphocholine-associated metabolism, remained comparatively stable across conditions.

These findings indicate that metabolic flexibility in GABAergic neurons is remodeled in a reaction-specific manner during disease progression rather than undergoing a globally coordinated contraction. Collectively, the corrected FVA results support selective remodeling of feasible metabolic states and heterogeneous redistribution of metabolic capabilities across the MCI–AD continuum.

### 3.8 Qualitative plausibility using independent metabolomic data

To evaluate the biological plausibility of the inferred metabolic states, pathway-level predictions derived from the contextualized GABAergic models were compared with independent metabolomic observations reported by Lysaker et al. (2025). Because the reference study quantified metabolite abundances and isotope-labeling patterns rather than intracellular metabolic fluxes, the comparison was performed qualitatively at the pathway level. Similarly, the external transcriptomic comparison with the SEA-AD dataset was intended to evaluate preservation of disease-associated transcriptional patterns rather than transcriptome-wide equivalence between datasets with substantially different biological and technical characteristics.

Several metabolic pathways predicted by the model exhibited directional agreement with experimentally reported alterations. In particular, altered flux variability in reactions associated with glutamine/glutamate metabolism, α-ketoglutarate metabolism, succinate-associated pathways, aspartate metabolism, malate–aspartate shuttle activity, and NAD-related metabolism was consistent with metabolomic and isotope-tracing evidence indicating alterations in central carbon metabolism, mitochondrial function, and neurotransmitter-associated pathways in APOE4 cellular models (Table 4).

Among the strongest concordances, the model predicted reaction-specific alterations in feasible flux ranges associated with α-ketoglutarate and succinate metabolism, consistent with experimentally observed perturbations in tricarboxylic acid (TCA) cycle activity and mitochondrial metabolism. Similarly, alterations predicted in aspartate-associated pathways were consistent with reported remodeling of malate–aspartate shuttle activity, a key mechanism linking cytosolic and mitochondrial metabolism.

The model also predicted alterations in metabolic flexibility within glutamine/glutamate-associated pathways, which is compatible with experimental observations of altered glutamate metabolism. In contrast, although changes in NAD-related metabolism were predicted by the model, the experimental study reported increased NAD and NADH labeling in APOE4 cells, suggesting that these observations may reflect compensatory metabolic responses rather than direct correspondence between metabolite abundance and intracellular flux states.

Importantly, no significant changes in lactate abundance were reported in the experimental dataset, and direct measurements of glutathione metabolism or GABA transport were not available. Consequently, these pathways were not considered direct validation targets and were excluded from the final concordance assessment.

Overall, the observed agreement supports the biological plausibility of the inferred metabolic states and suggests that the contextualized models capture biologically meaningful aspects of disease-associated metabolic remodeling. However, these comparisons should not be interpreted as formal validation because the external datasets differ substantially in genotype, brain region, cellular context, and experimental design.

The comparisons with independent metabolomic and transcriptomic datasets should be interpreted as qualitative assessments of biological plausibility rather than formal validation of the predicted metabolic states. The external datasets differ substantially from the present study with respect to genotype, brain region, cellular context, and experimental design. Consequently, both concordances and discrepancies likely reflect a combination of shared neurodegenerative mechanisms and context-dependent aspects of metabolic remodeling.

## 4 Discussion

### 4.1 Summary of key findings and conceptual advance

We present a genome-scale metabolic model (GEM) reconstructed from a GABAergic-enriched transcriptional component and contextualized along the mild cognitive impairment–Alzheimer’s disease (MCI–AD) continuum. Through the integration of transcriptomic deconvolution with constraint-based modeling, our results suggest progressive reaction-specific metabolic alterations in GABAergic neurons, characterized by alterations in energy metabolism, neurotransmitter cycling, and redox homeostasis. Beyond descriptive molecular alterations, the approach provides a systems-level framework linking transcriptomic alterations with predicted metabolic states generated through constraint-based modeling, supporting progressive remodeling of metabolic capacity and reaction-specific alterations in metabolic flexibility during disease progression.

A key contribution of this work is the integration of deconvolved transcriptomic profiles with constraint-based metabolic modeling to infer condition-specific metabolic phenotypes in GABAergic neurons. While previous studies have mainly characterized transcriptomic or molecular signatures in isolation, GEM-based approaches enable the integration of multi-omics data into a coherent systems-level representation of cellular function. However, most existing models remain generic and fail to capture the cellular specificity required to study complex tissues such as the brain. Our approach addresses a key limitation in the field and aligns with previous efforts demonstrating that tissue-specific genome-scale metabolic models (GEMs) can reveal functional metabolic alterations not evident from omics data alone (Baloni et al., 2020; Duarte et al., 2007; Moolamalla and Vinod, 2020).

Importantly, this conceptual framework is further supported by the structural organization of the reconstructed model, which is characterized by a predominance of transport and exchange reactions. The predominance of transport reactions is consistent with the high metabolic demands of GABAergic neurons and their dependence on continuous exchange of amino acids and energy substrates with neighboring astrocytes. The enrichment of amino acid, monocarboxylate, and mitochondrial transport systems further emphasizes the importance of neuron–astrocyte metabolic coupling and intracellular metabolite compartmentalization in sustaining inhibitory neurotransmission and metabolic homeostasis.

An important limitation of the reconstructed network is its predominance of transport and exchange reactions relative to mitochondrial and TCA-cycle-associated reactions. Consequently, the model is particularly suitable for investigating alterations in metabolite transport, neurotransmitter cycling, and intercompartmental metabolic interactions, whereas predictions regarding mitochondrial dysfunction should be interpreted as alterations in mitochondrial-associated metabolism rather than direct evidence of mitochondrial collapse.

This observation suggests that the metabolic maintenance of GABAergic neurons largely depends on intercompartmental metabolite exchange and on metabolic support provided by neighboring cells such as astrocytes. In this context, GABA synthesis depends directly on the availability of glutamate, which is converted into GABA through the action of glutamate decarboxylase enzymes (GAD1/GAD2). However, sustaining this metabolic cycle requires the active participation of astrocytes, which uptake GABA and glutamate from the extracellular space, convert them into glutamine, and subsequently transfer them back to neurons for neurotransmitter synthesis (Andersen, 2025; Chobanian et al., 2004).

Rather than providing a comprehensive characterization of all biological processes involved in Alzheimer’s disease, our findings demonstrate that the contextualized GABAergic genome-scale metabolic model captures biologically plausible metabolic alterations across interconnected pathways. For example, integrative GEM-based analyses have identified dysregulation of cholesterol and bile acid metabolism as key factors in Alzheimer’s disease pathology, including links with the gut–brain axis (Baloni et al., 2020). These observations are consistent with our model, which shows alterations in key metabolic pathways such as glycolysis, the Krebs cycle, and glutamate/GABA metabolism, suggesting that disease-associated alterations are distributed across interconnected metabolic pathways rather than being restricted to isolated processes.

Similarly, the observed metabolic alterations may be associated with neuronal dysfunction rather than merely reflecting downstream consequences of disease progression. Because the contextualized metabolic states are inferred from transcriptomic constraints, the present analyses cannot establish causal relationships between metabolic remodeling and neuronal dysfunction. Instead, the results suggest that transcriptional and metabolic alterations occur in parallel and may participate in interconnected processes during neurodegeneration. Accordingly, the reported metabolic signatures should be interpreted in the context of the modeling assumptions used for reconstruction and contextualization rather than as unique solutions to neuronal metabolism.

An additional methodological consideration relates to the identification of the GABAergic transcriptional component. Although component annotation was initially guided by canonical GABAergic markers, an orthogonal validation using an independent inhibitory-neuron marker panel that excluded GAD1, GAD2, and SLC32A1 consistently supported the biological identity of the selected component across disease stages. Nevertheless, because metabolic reconstruction was performed only for the selected GABAergic-enriched component, the potential influence of alternative component selection on downstream metabolic predictions remains an important methodological question for future studies.

In particular, the progressive alteration of Krebs cycle intermediates, such as α-ketoglutarate, suggests a potential link between metabolism and epigenetic regulation. It has been shown that metabolites such as α-ketoglutarate, succinate, and acetyl-CoA function as signaling molecules that regulate chromatin state and gene expression, thereby influencing cellular identity and function (Martínez-Reyes and Chandel, 2020). Likewise, previous GEM-based studies have been limited using bulk tissue data, which obscures the metabolic contributions of distinct cellular populations. This has allowed the observation that neuronal and glial cells exhibit highly specialized and interdependent metabolic programs, and that not considering this heterogeneity limits the interpretability of metabolic models (Kishk et al., 2022). This highlights the need to model cell types specifically in neurodegeneration when performing deconvolution and identifying and stratifying models into specific cell types.

Importantly, the metabolic alterations reported here should be interpreted within the context of the inferred GABAergic-enriched transcriptional component used for model reconstruction. Consequently, the predicted alterations do not represent global brain metabolism, but rather computationally inferred metabolic states associated with GABAergic-related transcriptional programs. This distinction is particularly relevant because neuronal subtypes exhibit distinct metabolic requirements and vulnerabilities during neurodegeneration.

The biological identity of the inferred GABAergic component was further supported through an independent validation using a 30-gene inhibitory-neuron signature that excluded GAD1, GAD2, and SLC32A1 (Supplementary Figure S6; Supplementary Table S6). The overall concordance between the independently enriched components and those selected for metabolic reconstruction indicates that the downstream metabolic analyses were supported by broader inhibitory-neuron transcriptional programs rather than being determined solely by the canonical marker genes initially used for component annotation.

Collectively, these findings suggest that metabolic dysfunction in GABAergic neurons extends beyond intracellular alterations and involves disruption of neuron–astrocyte metabolic coupling. Predicted changes affecting glutamate, glutamine, lactate, and redox-related pathways are consistent with impaired metabolic cooperation between these cell populations, a process essential for neurotransmitter recycling, energetic support, and maintenance of neuronal homeostasis. These observations reinforce the concept that neurodegeneration emerges from disturbances affecting interconnected metabolic networks rather than isolated biochemical pathways, highlighting the importance of considering cellular interactions and metabolic cross-talk when investigating disease mechanisms and identifying potential therapeutic targets.

Importantly, these metabolism-specific findings should be interpreted within the representational scope of the contextualized GEM. Accordingly, the pathways discussed throughout this work are intended as representative metabolic frameworks illustrating the predictive capability of the model, rather than an exhaustive ranking of the biological mechanisms underlying Alzheimer’s disease.

### 4.2 Metabolic remodeling associated with progressive GABAergic dysfunction

A progressive decrease in biomass-associated metabolic capacity and reaction-specific remodeling of feasible metabolic states was observed along the MCI–AD continuum (Figures 4A,B). Consistent with the flux balance analysis results, the contextualized models predicted lower optimal biomass fluxes and heterogeneous changes in feasible metabolic states across disease stages.

Consistent with the objective of evaluating the contextualized GEM, the following interpretation focuses on metabolic pathways directly represented within the reconstructed network and interrogated through FBA and FVA. This transition is reflected in the reaction-specific alterations in feasible fluxes through glycolysis, the TCA cycle, and oxidative phosphorylation, consistent with previously reported alterations in mitochondrial-associated metabolism during brain aging and Alzheimer’s disease, where decreased glucose utilization, ATP production, and overall bioenergetic efficiency are commonly observed (Na et al., 2025).

The resulting metabolic dysfunction may be further associated with alterations in key metabolic regulatory axes, particularly the NAD^+^–SIRT1 system, whose dysfunction compromises mitochondrial biogenesis, mitophagy, and cellular responses to energetic stress, ultimately promoting a chronic state of neuronal energy deficit (Fang et al., 2021).

This chronic energetic deficit may also be reinforced by oxidative damage affecting central metabolic pathways. In this regard, redox proteomics studies have demonstrated that oxidative stress can alter key enzymes involved in glycolysis and mitochondrial metabolism, including GAPDH, α-enolase, aconitase, creatine kinase, and ATP synthase. These oxidative modifications compromise glucose utilization and ATP production, thereby exacerbating bioenergetic dysfunction and impairing neurotransmission (Butterfield and Halliwell, 2019).

Similarly, the reduced feasible fluxes predicted in pathways associated with glutamate, glutamine, and GABA metabolism are compatible with alterations in the glutamate–glutamine cycle and neuron–astrocyte metabolic coupling. Human astrocytes play a central role in amino acid metabolism and neurotransmitter support, particularly through branched-chain amino acid metabolism, a process shown to be altered in Alzheimer’s disease models, thereby reducing the availability of essential metabolic precursors required for inhibitory neurotransmission (Salcedo et al., 2021).

Furthermore, neuronal metabolic dysfunction may not be exclusively an intracellular phenomenon, but may also reflect altered metabolic cooperation between cellular compartments. Indeed, brain metabolic organization depends on the integrated interaction between neurons, astrocytes, and the cerebral vasculature, where astrocytes provide lactate, regulate redox homeostasis, and contribute to the detoxification of reactive species (Shichkova et al., 2024).

Beyond the reduction in optimal biomass-associated flux values, flux variability analysis (FVA) revealed reaction-specific and stage-dependent changes in feasible flux ranges across several metabolic pathways (Figure 4B), including glutamine metabolism, lactate transport, glutamate-associated transport, lipid metabolism, and redox-related reactions. Within the constraint-based modeling framework, these results indicate selective remodeling of the feasible metabolic solution space, with different pathways exhibiting distinct patterns of flux redistribution across disease stages. Collectively, the corrected FVA results support reaction-specific metabolic remodeling rather than a uniform contraction of metabolic flexibility across all pathways.

Under physiological conditions, metabolic flexibility is associated with the ability of cells to redistribute metabolic fluxes and adapt to changing energetic demands, including transitions between glycolysis and oxidative phosphorylation (Bach and Nguyen, 2026). Consequently, the reaction-specific remodeling of feasible flux ranges predicted by the model suggests selective alterations in metabolic flexibility, potentially limiting the ability of some metabolic pathways to adapt to changing energetic and functional demands, potentially limiting the ability of GABAergic neurons to redistribute metabolic resources and adapt to changing energetic and functional demands. This reduction in metabolic adaptability may represent a previously underappreciated systems-level feature of neuronal vulnerability during early neurodegeneration. However, because FVA evaluates the range of feasible solutions permitted by the model constraints, these findings should be interpreted as computationally inferred properties of the contextualized metabolic networks rather than direct measurements of cellular adaptive capacity.

Under physiological conditions, metabolic flexibility has been associated with the ability of cells to redistribute metabolic fluxes in response to changing energetic demands, including transitions between glycolysis and oxidative phosphorylation (Bach and Nguyen, 2026). Within the context of constraint-based modeling, however, Flux Variability Analysis characterizes the range of feasible steady-state flux distributions permitted by the imposed model constraints rather than directly measuring biological adaptability. Therefore, the reaction-specific remodeling of feasible flux ranges observed in the contextualized models should be interpreted as selective changes in the computational solution space of the metabolic network. These alterations may reflect multiple factors, including changes in network redundancy, propagation of model constraints, the existence of alternative optimal flux distributions, and dependence on the selected objective function. Consequently, although the observed reaction-specific remodeling is consistent with altered metabolic organization during disease progression, it should not be interpreted as direct evidence of impaired neuronal adaptive capacity.

Metabolomic studies have shown that neurodegeneration is associated with dynamic alterations in energy metabolism and in the homeostasis of metabolites such as lactate and glutamate (Geiszler et al., 2018), highlighting the importance of metabolic adaptability for neuronal function. In this context, the present model suggests that disease progression may be accompanied by increasingly remodeled metabolic states characterized by selective pathway-specific constraints affecting energy metabolism, neurotransmitter-associated pathways, and redox homeostasis. It is important to note that the present model does not resolve the isoform-specific directionality of lactate dehydrogenase activity. In Recon3D, the LDH_L reaction aggregates multiple lactate dehydrogenase isoforms, including LDHA and LDHB, into a single reversible reaction. Consequently, the predicted reductions in LDH-associated fluxes and flux variability should be interpreted as a progressive restriction in the metabolic flexibility of lactate–pyruvate interconversion rather than direct evidence of reduced lactate production or reduced lactate utilization. This distinction is particularly relevant in the context of neuron–astrocyte metabolic coupling, where both lactate production and consumption contribute to metabolic homeostasis.

Importantly, these predictions represent computational properties of the contextualized metabolic models inferred under the selected optimization framework rather than direct measurements of neuronal metabolic adaptability or biological flexibility. Importantly, these predictions should be interpreted within the context of a transcriptomics-constrained modeling framework and therefore represent computationally inferred metabolic alterations rather than experimentally demonstrated mechanisms.

### 4.3 Metabolic remodeling and loss of inhibitory neurotransmission

Building upon the observed reduction in metabolic capacity and flexibility, our results indicate that metabolic dysfunction may contribute to impaired inhibitory neurotransmission in GABAergic neurons. Importantly, these findings specifically support a metabolic contribution to inhibitory neurotransmission within the scope of the contextualized GEM and should not be interpreted as encompassing the broader structural and electrophysiological determinants of synaptic dysfunction described in Alzheimer’s disease.

In particular, the progressive reduction of fluxes associated with glutamine, glutamate, and GABA metabolism, as observed in the contextualized model, suggests an alteration of the metabolic pathways necessary for the sustained synthesis and recycling of neurotransmitters. Under physiological conditions, GABA production depends on α-ketoglutarate derived from the Krebs cycle, as well as on glutamine supplied by astrocytes through tightly regulated transport systems. This neuron–astrocyte metabolic coupling is critical, as it regulates the synthesis and recycling of the neurotransmitter, directly determining presynaptic GABA levels and the efficacy of neuronal inhibition (Roth and Draguhn, 2012).

Likewise, GABA metabolism is intrinsically linked to cellular energy metabolism, since its degradation generates intermediates that re-enter the Krebs cycle, thereby establishing a bidirectional relationship between the bioenergetic state and neurotransmission (Ochoa-de la Paz et al., 2021). Consequently, alterations in central carbon metabolism may influence pathways associated with neurotransmitter homeostasis.

Considering this framework, the contextualized models predicted reduced feasible fluxes in TCA-related intermediates, glutamine fluxes, and transport-associated reactions, together with the transcriptional downregulation of key components of inhibitory signaling, including GABBR1 and GABBR2 (Figure 2A). These alterations are consistent with reduced availability of metabolic precursors required for inhibitory neurotransmission, potentially limiting metabolic support for inhibitory neurotransmission. This is further supported by the progressive decline in biological processes associated with GABAergic synaptic transmission and neurotransmitter regulation observed in the functional enrichment analysis (Figure 2B).

Importantly, these findings suggest that metabolic dysfunction may contribute to alterations in inhibitory neurotransmission during disease progression. In particular, reduced fluxes associated with neurotransmitter metabolism and energy-related pathways were consistent with impaired metabolic support for synaptic function and altered excitatory–inhibitory balance. Together, these results support a systems-level association between bioenergetic alterations, disrupted metabolic interactions, and neuronal dysfunction (Wang et al., 2025).

### 4.4 Transport-dependent metabolic coupling underlies neurotransmitter, redox, and lipid dysregulation in GABAergic neurons

The available evidence indicates that transport processes constitute a central axis in the regulation of metabolic fluxes associated with glutamine, glutamate, and GABA, as well as in the maintenance of redox and lipid homeostasis. This provides a mechanistic basis for interpreting the patterns observed in our model.

Primarily, it has been demonstrated that the glutamate/GABA–glutamine cycle critically depends on a highly coordinated system of intercellular transport between neurons and astrocytes. Excitatory amino acid transporters (EAATs) mediate the uptake of glutamate from the synaptic space into astrocytes, followed by its conversion into glutamine and subsequent export via sodium-coupled neutral amino acid transporters (SNAT) to neurons, where it is reused for the synthesis of glutamate and GABA ((Andersen, 2025; Bak et al., 2006). This process is not only essential for inhibitory neurotransmission but also tightly coupled to the tricarboxylic acid (TCA) cycle through intermediates such as α-ketoglutarate, such that any limitation in transport directly alters neurotransmitter-related metabolic fluxes.

In addition, the expression and activity of specific transporters, including EAAT, VGLUT, and glutamine transport systems, regulate the availability of carbon and nitrogen sources in neurons, thereby affecting both neurotransmitter synthesis and broader metabolic pathways (Zhang et al., 2024). In this context, the predominance of transport reactions observed in the reconstructed model supports the prominence of transport-associated reactions within the reconstructed network.

Furthermore, experimental evidence has shown that under pathological conditions such as Alzheimer’s disease, astrocytes exhibit a reduced capacity to metabolize branched-chain amino acids and to generate glutamate and glutamine, thereby limiting the supply of essential metabolic precursors for neuronal neurotransmission (Salcedo et al., 2021). This suggests that the reductions in glutamine, glutamate, and GABA fluxes observed in our model are not solely the result of intrinsic neuronal dysfunction, but also reflect impaired neuron–astrocyte metabolic coupling mediated by transport processes.

From a redox perspective, glutamate acts as a key precursor for glutathione (GSH) synthesis. Therefore, reduced availability of glutamate, together with decreased NADPH production, negatively impacts antioxidant capacity. In this sense, the reduction in glutathione-associated fluxes observed in our model can be interpreted as a direct consequence of impaired substrate transport and metabolic coupling.

Similarly, lipid metabolism must be considered within this transport-dependent framework. Neurons exhibit a limited capacity for *de novo* cholesterol synthesis and therefore depend heavily on astrocyte-derived lipid transport, including cholesterol and phospholipids such as glycerophosphocholine (Shichkova et al., 2024). Alterations in these transport processes can compromise membrane integrity, synaptic signaling, and structural stability. This is consistent with the reduction in fluxes associated with cholesterol and glycerophosphocholine observed in our model.

Collectively, these findings indicate that disruption of transport-associated interactions may contribute to reduced metabolic coordination across pathways.

### 4.5 Systems-level integration of metabolic dysfunction

Beyond alterations in individual pathways, the contextualized GABAergic GEM supports a systems-level representation of coordinated metabolic remodeling during disease progression. This perspective is consistent with emerging systems biology approaches that conceptualize neurodegenerative diseases as perturbations of complex, multilayered biological networks (Baloni et al., 2021).

In this context, the alterations observed in energy metabolism, metabolite transport, redox balance, neurotransmitter cycling, and lipid homeostasis should not be interpreted as isolated defects, but rather as interconnected components of a progressive disruption of metabolic integration in GABAergic neurons.

A central feature of this metabolic dysfunction is the bioenergetic deficit, reflected in the reduction of flux through glycolysis, the TCA cycle, and oxidative phosphorylation. This energetic deterioration is particularly critical in the brain, where metabolism operates as a highly coordinated network among cell types and subcellular compartments, requiring continuous energy supply and efficient metabolite exchange (Madrer et al., 2025).

Importantly, reduced energy availability directly impacts transport capacity, due to the strong dependence of transport processes on ATP and electrochemical gradients. As a result, the reduction in transport fluxes observed in our model limits the availability of key substrates such as glutamine and lactate, thereby impairing metabolic communication between cellular compartments.

This restriction propagates across interconnected pathways. The reduced availability of glutamine and TCA intermediates such as α-ketoglutarate limits glutamate and GABA synthesis, establishing a direct link between bioenergetic failure and neurotransmission dysfunction. At the same time, reduced NADPH-generating capacity and impaired glutathione metabolism compromise redox homeostasis, increasing susceptibility to oxidative stress.

Additionally, alterations in lipid metabolism, including reduced fluxes associated with cholesterol and glycerophosphocholine, suggest impaired membrane maintenance and synaptic integrity. These findings are consistent with evidence linking metabolic dysfunction to structural alterations in neuronal networks, where energy demand and functional organization are tightly coupled.

**FIGURE 5:**
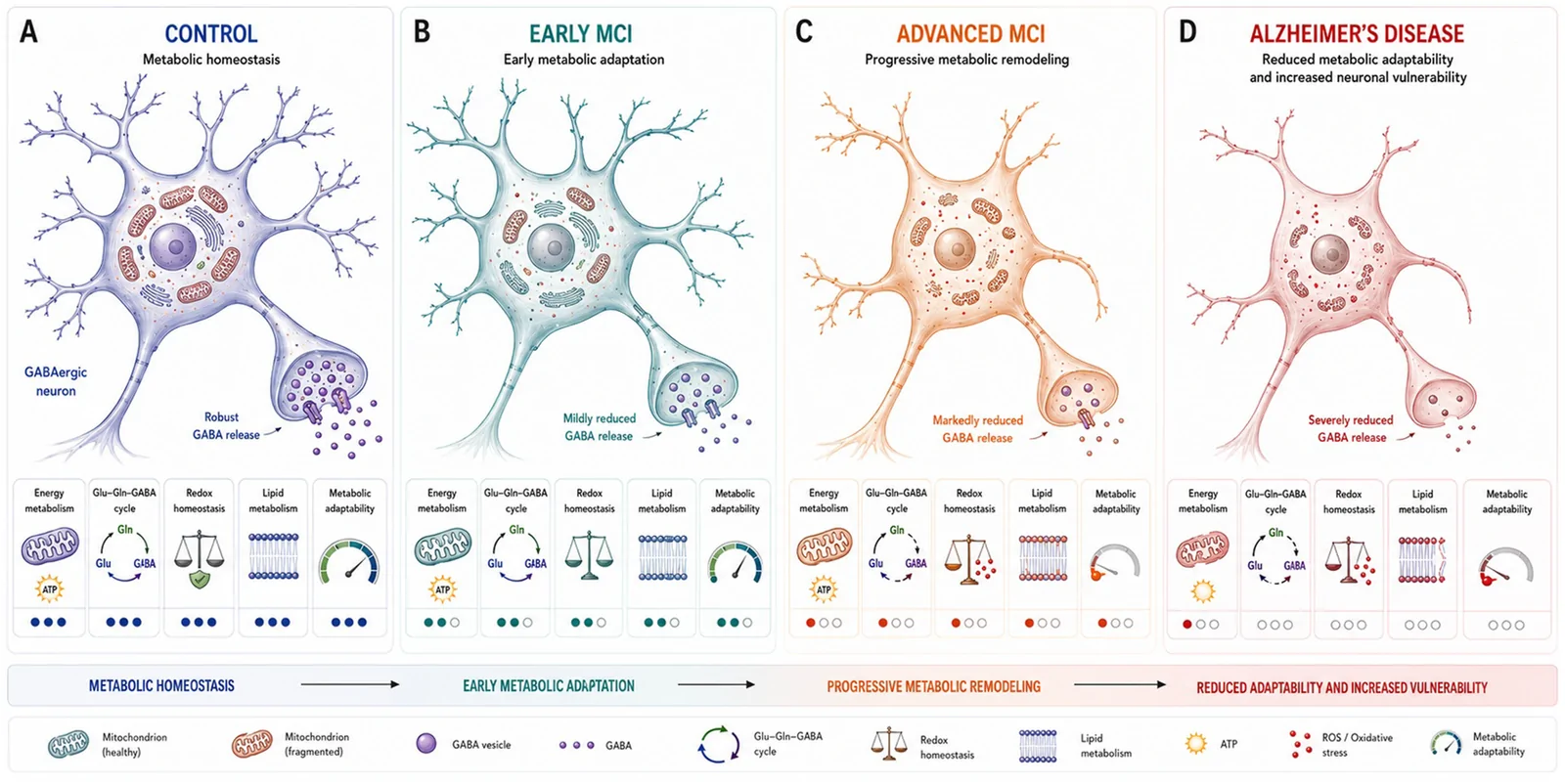
Conceptual model of progressive metabolic remodeling in GABAergic neurons across the mild cognitive impairment–Alzheimer’s disease continuum. The figure summarizes the inferred biological changes in GABAergic neurons across four disease stages based on the contextualized genome-scale metabolic models. **(A)** Control: GABAergic neurons exhibit metabolic homeostasis, characterized by preserved energy metabolism, efficient glutamate–glutamine–GABA cycling, balanced redox homeostasis, maintained lipid metabolism, high metabolic adaptability, and robust inhibitory neurotransmission. **(B)** E-MCI: Early metabolic adaptation is characterized by subtle mitochondrial alterations, a mild reduction in ATP availability, initial remodeling of the glutamate–glutamine–GABA cycle, early oxidative imbalance, and a slight decrease in inhibitory neurotransmitter release while overall metabolic organization remains largely preserved. **(C)** A-MCI: Progressive metabolic remodeling is associated with impaired mitochondrial function, reduced energy production, disruption of glutamate–glutamine–GABA metabolism, increased oxidative stress, alterations in lipid metabolism, diminished inhibitory neurotransmission, and reduced metabolic adaptability. **(D)** AD: GABAergic neurons display a metabolically vulnerable phenotype characterized by persistent mitochondrial dysfunction, low ATP availability, disruption of neurotransmitter cycling, sustained oxidative imbalance, altered lipid homeostasis, markedly reduced GABA release, and minimal metabolic adaptability. Together, these stages illustrate a progressive transition from metabolic homeostasis to reaction-specific metabolic remodeling and reduced metabolic flexibility, culminating in increased neuronal vulnerability during Alzheimer’s disease progression.

Collectively, these findings support a systems-level interpretation in which progressive alterations in energy metabolism, transport-associated processes, redox balance, and neurotransmitter cycling are closely interconnected during disease progression. Reduced metabolic fluxes and transport capacity may limit substrate availability and synaptic metabolic support, contributing to widespread dysfunction in GABAergic neurons across the MCI–AD continuum. This integrated framework is summarized in Figure 5 (Angarita-Rodríguez et al., 2024).

### 4.6 Early metabolic alterations in MCI

A key insight of this study is that metabolic dysfunction is already detectable during the early stages of mild cognitive impairment, prior to the onset of full Alzheimer’s disease. Rather than a discrete transition, our results reveal a gradual metabolic trajectory from control to E-MCI, A-MCI, and AD, characterized by progressive reductions in metabolic fluxes, transport capacity, and metabolic flexibility.

This observation is consistent with longitudinal metabolomics studies showing that metabolic alterations emerge in prodromal stages and persist throughout disease progression. Indeed, early changes in membrane lipids, energy metabolism, and oxidative stress markers have been reported in MCI cohorts that later progress to Alzheimer’s disease (Marella et al., 2025; Orešič et al., 2011).

The intermediate behavior observed in E-MCI and A-MCI is particularly relevant, as it indicates that metabolic alterations were also detected in early disease stages. Instead, key pathways—including glycolysis, the TCA cycle, lactate metabolism, glutamine/glutamate/GABA metabolism, NADPH-generating reactions, and glutathione metabolism-already exhibit partial impairment during prodromal phases.

From a systems perspective, these findings suggest that metabolic alterations emerge during prodromal stages and become progressively more pronounced across disease progression. However, these interpretations should be considered in light of the transcriptomics-based nature of the reconstruction, the absence of direct functional validation, and the lack of proteomic, fluxomic, and enzyme activity measurements. Consequently, the predicted metabolic states should be interpreted as computationally inferred representations of disease-associated metabolic alterations rather than experimentally demonstrated mechanisms.

### 4.7 Model validation and biological relevance

The biological validity of the GABAergic neuron-specific genome-scale metabolic model was evaluated through a multi-layer validation strategy integrating transcriptomic and metabolomic evidence, thereby strengthening the robustness of the proposed framework. This integrative approach is consistent with established principles of constraint-based modelling (Bordbar et al., 2014).

At the transcriptomic level, validation was performed in three complementary steps. First, an independent inhibitory-neuron marker signature excluding GAD1, GAD2, and SLC32A1 confirmed the biological identity of the inferred CDSeq component (Supplementary Figure S6; Supplementary Table S6). Second, canonical GABAergic markers together with inhibitory neuronal populations from the SEA-AD atlas further supported the cellular identity of the selected component (Supplementary Figure S4). Third, disease-stage transcriptional profiles were compared with harmonized SEA-AD pseudobulk profiles (Figure 3D), demonstrating moderate but consistent concordance across equivalent disease stages. This analysis revealed moderate but consistent concordance across equivalent clinical conditions, with correlation coefficients of r = 0.42 for Control–Control, r = 0.52 for E-MCI–E-MCI, r = 0.54 for A-MCI–A-MCI, and r = 0.45 for AD–AD. In contrast, non-equivalent comparisons exhibited lower correlations, supporting the partial preservation of disease-associated neuronal transcriptional trends across independent datasets rather than reflecting nonspecific signal overlap.

Importantly, the moderate magnitude of these correlations likely reflects biological and technical heterogeneity between datasets, including regional specificity (middle temporal gyrus versus hippocampal-derived datasets), differences in sequencing platforms, and the intrinsic complexity of deconvolution-based inference. Indeed, the observed pattern of stage-consistent correlations, together with the gradual transitions across intermediate conditions, supports the capacity of the model to capture biologically meaningful transcriptional dynamics associated with disease progression.

Because the primary dataset originated from hippocampal CA1 tissue whereas the validation dataset was derived from the middle temporal gyrus, the observed correlations may reflect shared neurodegenerative transcriptional programs and regional transcriptomic overlap rather than direct validation of an identical neuronal population.

At the functional level, flux variability analysis (FVA) revealed reaction-specific and stage-dependent changes in feasible flux ranges across pathways associated with glutamine metabolism, lactate and GABA transport, glutamate-associated transport, lipid metabolism, and redox-related processes. Within the constraint-based modeling framework, these alterations indicate selective remodeling of the feasible metabolic solution space, with different pathways exhibiting distinct patterns of flux redistribution across disease stages. Collectively, the corrected FVA results support reaction-specific metabolic remodeling rather than a uniform contraction of metabolic flexibility across all pathways.

It is important to emphasize that these pathways were not intended to represent an exhaustive ranking of the biological processes affected during Alzheimer’s disease. Rather, they constitute representative metabolic frameworks that were directly interrogated using the contextualized GABAergic genome-scale metabolic model and flux variability analysis. These pathways were selected because they are explicitly represented within the metabolic network and because independent experimental evidence is available to qualitatively assess the biological plausibility of the predicted metabolic alterations. Consequently, the present analysis should be interpreted as an evaluation of the predictive capacity of the contextualized GEM to capture disease-associated metabolic remodeling, rather than as a comprehensive assessment of all pathological mechanisms underlying Alzheimer’s disease.

To assess the biological plausibility of the inferred metabolic states, pathway-level predictions generated by the contextualized GABAergic models were compared with independent metabolomic and isotope-tracing observations reported by Lysaker et al. (2025). Although metabolite abundances and intracellular fluxes represent distinct biological quantities, several pathways identified by the model exhibited directional agreement with experimentally reported alterations. In particular, pathways associated with glutamine/glutamate metabolism, α-ketoglutarate metabolism, succinate-associated metabolism, aspartate metabolism, malate–aspartate shuttle activity, and NAD-related metabolism showed qualitative consistency with experimental observations of altered central carbon metabolism, mitochondrial function, and neurotransmitter-associated processes. Importantly, this agreement should be interpreted as pathway-level consistency rather than direct validation of predicted flux values. The convergence observed across multiple independent metabolic processes nevertheless supports the biological plausibility of the inferred metabolic states and highlights the utility of the model as a systems-level framework for investigating disease-associated metabolic alterations during neurodegeneration.

Although synaptic dysfunction, impaired neuronal connectivity, and electrophysiological alterations are widely recognized as central features of Alzheimer’s disease, many of these processes involve mechanisms that extend beyond the scope of constraint-based metabolic modeling, including receptor trafficking, synaptic vesicle dynamics, dendritic remodeling, and neuronal excitability. In contrast, the present study specifically addresses metabolic network organization through a contextualized GABAergic GEM. Therefore, the biological insights generated by the model should be viewed as complementary to, rather than replacing, the extensive body of literature describing non-metabolic aspects of neuronal dysfunction in Alzheimer’s disease.

### 4.8 Limitations and future directions

Despite the strengths of the proposed model, several limitations should be considered when interpreting the results.

First, the reconstruction of the GABAergic-enriched metabolic model relied primarily on transcriptomic data. Although gene expression provides valuable insights into cellular states, it does not necessarily correlate directly with enzymatic activity, protein abundance, or post-translational regulation. Consequently, the inferred metabolic fluxes represent computational estimates of feasible metabolic states and may not fully reflect the actual physiological behavior of GABAergic neurons. This limitation is inherent to most transcriptomics-driven GEM reconstructions and highlights the need for integrating additional layers of regulation. Similarly, because several reactions in Recon3D aggregate multiple enzyme isoforms into single reversible reactions, the model cannot infer isoform-specific catalytic directionality, and therefore some pathway interpretations should be restricted to reaction-level metabolic capacities.

An additional limitation is the relatively small size of the transcriptomic cohort used for model contextualization. The GSE28146 dataset included *postmortem* hippocampal CA1 samples from 30 individuals distributed across four clinical groups (Control, *n* = 8; Early MCI, *n* = 7; Advanced MCI, *n* = 8; Alzheimer’s disease, *n* = 7). Because *postmortem* brain transcriptomic datasets are inherently difficult to obtain, studies often involve relatively limited sample sizes. Nevertheless, the reduced number of individuals per group may decrease statistical power and increase susceptibility to inter-individual biological variability. Therefore, the metabolic alterations inferred by the contextualized GEM should be interpreted as hypothesis-generating predictions that warrant confirmation in larger, independent, and preferably larger, independent cohorts and, ideally, longitudinal studies incorporating cell-type-resolved transcriptomic data.

Second, the use of Recon3D as the reference metabolic network introduces inherent constraints, as this model was not specifically designed for neuronal biology but rather represents a generalized reconstruction of human metabolism (Brunk et al., 2018; Ebrahim et al., 2016). As a result, certain neuron-specific pathways and cell-type-specific metabolic features may be incompletely represented. Furthermore, because only a fraction of the reactions contained in the original Recon3D network could be associated with transcriptomic evidence during contextualization, a substantial proportion of the potential metabolic activity represented in the generic network was not incorporated into the final condition-specific models. This limitation arises both from the incomplete correspondence between gene expression and metabolic activity and from the absence of neuron-specific pathways in the reference reconstruction. Although contextualization and manual curation partially mitigated these limitations, the resulting models should be interpreted as constrained representations of GABAergic-enriched metabolic states rather than exhaustive reconstructions of neuronal metabolism.

Third, an additional limitation concerns the definition of the extracellular nutritional environment used to constrain the model. Nutritional exchanges were defined using a DMEM/F12-derived formulation supplemented with GlutaMAX, BDNF and FBS to maintain methodological consistency with the parental reconstruction from which the present neuronal model was developed. Although serum-free formulations such as BrainPhys provide a more physiologically relevant extracellular environment for experimental neuronal cultures, they were primarily designed to preserve neuronal electrophysiological properties, including synaptic activity and functional maturation, processes that are not explicitly represented within the present constraint-based metabolic framework. Consequently, while the nutritional composition may influence the feasible exchange space of the model, it is unlikely to directly affect electrophysiological features that are beyond the scope of genome-scale metabolic modeling. Nevertheless, future refinements would benefit from incorporating quantitatively defined BrainPhys- or cerebrospinal fluid-based metabolic constraints as these formulations become more comprehensively characterized for GEM applications.

Furthermore, the adaptation of microarray-derived transcriptomic data for deconvolution analysis using CDSeq may introduce biases associated with differences between sequencing platforms, as well as limitations intrinsic to microarray technology, including reduced dynamic range and probe specificity. Additionally, CDSeq was originally developed for RNA-seq data and was adapted here for microarray-derived expression profiles. Although external validation supported the biological plausibility of the inferred transcriptional component, platform-specific biases may influence deconvolution accuracy and the resulting metabolic contextualization. The external validation dataset was derived from the middle temporal gyrus, whereas the primary dataset corresponds to the hippocampal CA1 region, which may contribute to the moderate correlation values observed between datasets.

In addition, although sensitivity analyses demonstrated that the inferred GABAergic-enriched component was robust across different values of *num_cell_types*, deconvolution of a relatively minor neuronal population from bulk hippocampal transcriptomic data remains an indirect computational inference. Consequently, the inferred cell-type-specific expression profiles should be interpreted as probabilistic representations of GABAergic transcriptional states rather than direct measurements of isolated neuronal populations. Accordingly, the reconstructed metabolic models represent GABAergic-enriched metabolic states inferred from transcriptomic data and should not be interpreted as exhaustive representations of a pure neuronal subtype.

From a modeling perspective, both flux balance analysis (FBA) and flux variability analysis (FVA) rely on steady-state assumptions, which limit the ability of the model to capture temporal dynamics and transient metabolic states. In addition, FVA characterizes the range of feasible solutions permitted by the imposed constraints and therefore should not be interpreted as a direct measurement of cellular metabolic adaptability. Reductions in feasible flux variability may reflect altered network redundancy, propagation of model constraints, the existence of multiple optimal solutions, or dependence on the selected objective function, rather than exclusively indicating reduced biological adaptability. Consequently, the model represents an approximation of feasible metabolic states rather than dynamic cellular responses.

Furthermore, while the model incorporates transcriptional information to constrain metabolic fluxes, it does not explicitly account for regulatory mechanisms such as signaling pathways, enzyme kinetics, protein turnover, or spatial compartmentalization beyond the predefined structure of the network. These factors may play critical roles in shaping metabolic responses during neurodegeneration.

Although sensitivity analyses supported the stability of the principal pathway-level conclusions, the present study further evaluated model robustness through complementary perturbation analyses of both the biomass formulation (Table 2) and global reaction-bound constraints (Supplementary Figure S1). In both analyses, moderate perturbations preserved the principal metabolic trends and the relative ordering of disease stages, indicating that the main biological conclusions are robust to these sources of modeling uncertainty. Nevertheless, additional sources of uncertainty remain inherent to transcriptomics-constrained genome-scale metabolic reconstruction, including alternative nutrient constraints, uncertainty in GPR associations, transcriptomic threshold selection during model contextualization, uncertainty associated with transcriptomic deconvolution, and Exp2Flux parameterization. Comprehensive evaluation of these additional modeling assumptions would repeated reconstruction and contextualization of the genome-scale metabolic models under multiple alternative parameterizations. Such analyses would further strengthen confidence in the predicted metabolic signatures and represent an important direction for future methodological studies. Likewise, the biomass objective function represents an additional source of uncertainty because neurons are post-mitotic cells whose primary metabolic objective is maintenance rather than proliferation. Accordingly, the neuron-specific biomass reaction implemented here should be interpreted as a computational surrogate for maintenance-associated metabolic demands rather than a representation of cellular growth, and alternative biomass formulations could still influence the absolute magnitude of predicted fluxes.

Finally, while comparison with independent metabolomic observations provided qualitative support for the inferred metabolic states, metabolite abundances and intracellular fluxes are fundamentally distinct biological quantities. Therefore, the observed agreement should be interpreted as pathway-level consistency rather than direct validation of model-predicted flux values. Importantly, no direct intracellular metabolic flux measurements were performed in the analyzed human hippocampal samples, and no isotope-tracing or fluxomic datasets were available to experimentally validate the predicted flux distributions. Consequently, the predicted metabolic alterations should be interpreted as hypothesis-generating computational inferences that require future experimental validation.

More broadly, all flux values and feasible flux ranges reported in this study remain computational inferences derived from transcriptomic constraints, network stoichiometry, the selected objective function, and the imposed reaction bounds. We did not perform direct measurements of intracellular metabolic fluxes in the analyzed human hippocampal samples, nor were isotope-tracing or fluxomic experiments available to validate the predicted flux distributions. Therefore, the reported alterations should not be interpreted as direct mechanistic confirmation of metabolic dysfunction. Rather, they represent hypothesis-generating predictions that identify candidate pathways and reaction-level metabolic alterations for future experimental evaluation.

A central model-derived finding of this study is a predicted progression of reaction-specific alterations in metabolic flexibility across the MCI–AD continuum, accompanied by changes in energy metabolism, neurotransmitter cycling, and neuron–astrocyte metabolic interactions. By integrating cell-type-specific transcriptomic information with genome-scale metabolic modeling, this work provides a systems-level framework for understanding how metabolic dysfunction contributes to the selective vulnerability of GABAergic neurons during the progression from mild cognitive impairment to Alzheimer’s disease. Beyond characterizing disease-associated metabolic alterations, this framework provides a basis for generating hypotheses regarding candidate metabolic biomarkers and metabolic pathways of potential therapeutic relevance. These computationally prioritized pathways should not be interpreted as validated therapeutic targets but rather as candidates for future experimental investigation.

Future studies should aim to address these limitations by integrating complementary multi-omics approaches, including spatial transcriptomics, single-cell metabolomics, metabolomics, proteomics, phosphoproteomics, and isotope-tracing experiments. In addition, validation in patient-derived induced pluripotent stem cell (iPSC) models and larger longitudinal cohorts will be essential to experimentally evaluate the computationally inferred metabolic alterations and characterize the temporal evolution of metabolic remodeling during disease progression. Furthermore, experimental validation using patient-derived neuronal systems, such as induced pluripotent stem cell (iPSC)-derived neurons, and the application of emerging single-cell metabolomics approaches will be essential to evaluate the predicted metabolic alterations and identify potential therapeutic targets. Incorporating dynamic modeling frameworks and time-resolved data could further enhance the predictive capacity of GEM-based approaches in neurodegeneration.

Finally, future studies should evaluate the proposed modeling framework in larger, independent, and longitudinal patient cohorts to determine how metabolic network remodeling evolves throughout disease progression. Longitudinal studies are essential for characterizing the temporal dynamics of biomarker changes and disease progression, providing insights that cannot be obtained from cross-sectional analyses alone (Jack et al., 2018; Marella et al., 2025). Integrating longitudinal transcriptomic, metabolomic, and fluxomic measurements with genome-scale metabolic modeling could further improve the biological interpretation, temporal robustness, and clinical relevance of the inferred metabolic states.

## Data Availability

The original contributions presented in the study are included in the article/Supplementary Material, further inquiries can be directed to the corresponding author.
