# Supplementary material for: Integrative genome-scale metabolic model of GABAergic neurons reveals metabolic signatures across the mild cognitive impairment – Alzheimer disease continuum
Source: Front Syst Biol. 2026 Sep 3;6:1897648. doi: 10.3389/fsysb.2026.1897648 (PMC13581822; doi:10.3389/fsysb.2026.1897648)
Supplement: Supplementary file 1 [file Supplementaryfile1.docx]

**Supplementary Figure S1. Sensitivity analysis of neuronal metabolic predictions to global reaction-bound perturbation.** Flux values of the objective function obtained from the context-specific neuronal metabolic models after uniformly contracting (−20%) or expanding (+20%) all reaction bounds relative to the original model. Although the absolute optimal objective flux varied proportionally with the imposed perturbation, the relative ranking of disease stages remained unchanged (Control > E-MCI > A-MCI > AD) across all scenarios. These results indicate that the principal metabolic conclusions are robust to moderate uncertainty in reaction-capacity constraints and are not driven by a specific parameterization of the reaction bounds.
