# Supplementary material for: Integrative genome-scale metabolic model of GABAergic neurons reveals metabolic signatures across the mild cognitive impairment – Alzheimer disease continuum
Source: Front Syst Biol. 2026 Sep 3;6:1897648. doi: 10.3389/fsysb.2026.1897648 (PMC13581822; doi:10.3389/fsysb.2026.1897648)
Supplement: Supplementary file 2 [file Supplementaryfile2.docx]

**Supplementary Figure S2. Age distribution across the clinical groups included in the study cohort.** Box-and-whisker plots showing the age distribution of individuals classified as Control (n = 8), early mild cognitive impairment (E-MCI, n = 7), advanced mild cognitive impairment (A-MCI, n = 8), and Alzheimer's disease (AD, n = 7). Boxes represent the interquartile range (IQR), the central line indicates the median, whiskers denote the minimum and maximum values, and individual dots correspond to each participant. Group differences were evaluated using a non-parametric Kruskal–Wallis One-Way ANOVA test. No significant differences in age were observed among the four clinical groups (χ²_(3, 26)_ = 5.858, p = 0.119). Nevertheless, pairwise *post-hoc* comparisons were performed using Mann–Whitney U tests with Bonferroni corrections; none of the pairwise comparisons were significant. Results show that groups were not different in age, minimizing the potential confounding effect of age in subsequent analyses.
