# Supplementary material for: Integrative genome-scale metabolic model of GABAergic neurons reveals metabolic signatures across the mild cognitive impairment – Alzheimer disease continuum
Source: Front Syst Biol. 2026 Sep 3;6:1897648. doi: 10.3389/fsysb.2026.1897648 (PMC13581822; doi:10.3389/fsysb.2026.1897648)
Supplement: Supplementary file 3 [file Supplementaryfile3.docx]

**Supplementary Figure S3.** Robustness analyses supporting the selection of the number of latent transcriptional components (num_cell_types). (A) Elbow plot showing the reconstruction error as a function of the number of latent components (k). The decrease in reconstruction error markedly slows beyond k = 6, indicating diminishing returns in model fit. (B) Mean silhouette coefficient across values of k, showing maximal component separability at k = 6. (C) Cosine similarity between the GABAergic-enriched component identified at each value of k and the component obtained at k = 6. The high similarity observed across all tested values demonstrates that the identification of the GABAergic transcriptional component is robust to the choice of num_cell_types. The dashed red line indicates the value used in the main analyses (k = 6).
