# Supplementary material for: Integrative genome-scale metabolic model of GABAergic neurons reveals metabolic signatures across the mild cognitive impairment – Alzheimer disease continuum
Source: Front Syst Biol. 2026 Sep 3;6:1897648. doi: 10.3389/fsysb.2026.1897648 (PMC13581822; doi:10.3389/fsysb.2026.1897648)
Supplement: Supplementary file 4 [file Supplementaryfile4.docx]

**Supplementary Figure S4. Stage-matched external concordance of GABAergic transcriptional profiles.** Points represent Spearman correlation coefficients between stage-matched deconvolved GABAergic transcriptional components from GSE28146 and GABAergic profiles from the independent SEA-AD dataset. Error bars represent 95% bootstrap confidence intervals.
