# Supplementary material for: Integrative genome-scale metabolic model of GABAergic neurons reveals metabolic signatures across the mild cognitive impairment – Alzheimer disease continuum
Source: Front Syst Biol. 2026 Sep 3;6:1897648. doi: 10.3389/fsysb.2026.1897648 (PMC13581822; doi:10.3389/fsysb.2026.1897648)
Supplement: Supplementary file 5 [file Supplementaryfile5.docx]

**Supplementary Figure S5. Functional characterization of transport reactions in the reconstructed GABAergic neuron genome-scale metabolic model.** **(A)** Distribution of transport reactions according to their major functional categories within the reconstructed network. Amino acid transport reactions constituted the largest fraction of transport-associated reactions, followed by mitochondrial carrier reactions and monocarboxylate transport processes. **(B)** Breakdown of amino acid transport reactions according to substrate class, highlighting the predominance of transport systems associated with glutamine, branched-chain amino acids, aromatic amino acids, glycine, and serine. The overall organization of transport reactions indicates that the reconstructed GABAergic metabolic network is highly dependent on extracellular metabolite exchange and intracellular compartmentalization of metabolites involved in neurotransmitter synthesis, energy metabolism, redox homeostasis, and neuron–astrocyte metabolic coupling.
