# Supplementary material for: Integrative genome-scale metabolic model of GABAergic neurons reveals metabolic signatures across the mild cognitive impairment – Alzheimer disease continuum
Source: Front Syst Biol. 2026 Sep 3;6:1897648. doi: 10.3389/fsysb.2026.1897648 (PMC13581822; doi:10.3389/fsysb.2026.1897648)
Supplement: Supplementary file 6 [file Supplementaryfile6.docx]

**Supplementary Figure S6. Independent validation of the selected CDSeq GABAergic latent components using an independent inhibitory-neuron gene signature.**Rank-based enrichment (area under the Mann–Whitney enrichment curve, AUC) of an independent inhibitory-neuron gene signature was evaluated for each of the six latent CDSeq components across the four disease stages (Control, E-MCI, A-MCI and AD). The independent marker set comprised 30 canonical inhibitory-neuron markers:GPHN, SLC6A1, ABAT, GABRA1, GABRA2, GABRA3, GABRA4, GABRB1, GABRB2, GABRB3, GABRG1, GABRG2, GABRG3, GABBR1, GABBR2, GABRD, GABRE, GABRQ, SST, RELN, VIP, PVALB, CALB1, CALB2, LHX6, DLX1, DLX2, ERBB4, CCK and KCNIP1. To avoid circular component annotation, GAD1, GAD2 and SLC32A1, which were used during the initial biological annotation of the CDSeq components, were intentionally excluded from this validation. Higher AUC values indicate stronger enrichment of the independent inhibitory-neuron signature within each latent component. ★ indicates the component with the highest independent-marker enrichment within each disease stage, whereas ○ denotes the component selected for the metabolic reconstruction. Statistical significance was assessed using one-sided Mann–Whitney tests followed by Benjamini–Hochberg false-discovery rate (FDR) correction.
