# Supplementary material for: Integrative genome-scale metabolic model of GABAergic neurons reveals metabolic signatures across the mild cognitive impairment – Alzheimer disease continuum
Source: Front Syst Biol. 2026 Sep 3;6:1897648. doi: 10.3389/fsysb.2026.1897648 (PMC13581822; doi:10.3389/fsysb.2026.1897648)
Supplement: Supplementary file 7 [file Table5.docx]

**Supplementary Table S5.** Major classes of transport reactions represented in the reconstructed GABAergic neuron metabolic model and their biological relevance.

| **Transport category** | **Representative metabolites** | **Biological relevance** |
| --- | --- | --- |
| Amino acid transport | Glutamine, glycine, serine, alanine, leucine, isoleucine, valine, phenylalanine, tryptophan, methionine, asparagine | Neurotransmitter synthesis, anaplerosis, redox balance, neuron–astrocyte metabolic coupling |
| Cationic amino acid transport | Arginine, lysine, ornithine, citrulline | Polyamine synthesis, nitric oxide metabolism, neurotransmitter regulation |
| Monocarboxylate transport | Lactate, pyruvate, acetate, ketone bodies | Energy metabolism, lactate shuttle, metabolic flexibility |
| Mitochondrial carrier reactions | Citrate, α-ketoglutarate, succinate, malate, fumarate | TCA cycle maintenance, redox homeostasis, biosynthetic precursor exchange |
| Vesicular transport | Cholesterol, amino acids and other metabolites | Intracellular trafficking and membrane dynamics |
| Nucleotide transport | ATP, orotate, pantothenate | Energy balance and cofactor biosynthesis |
