# Supplementary material for: Integrative genome-scale metabolic model of GABAergic neurons reveals metabolic signatures across the mild cognitive impairment – Alzheimer disease continuum
Source: Front Syst Biol. 2026 Sep 3;6:1897648. doi: 10.3389/fsysb.2026.1897648 (PMC13581822; doi:10.3389/fsysb.2026.1897648)
Supplement: Supplementary file 8 [file Table6.docx]

**Supplementary Table S6. Summary of independent validation of CDSeq component annotation using an independent GABAergic marker set**

| **Disease stage** | **Originally selected component** | **Independent-marker enrichment (AUC)** | **FDR-adjusted q-value** | **Rank among six components** | **Interpretation** |
| --- | --- | --- | --- | --- | --- |
| **Control** | C2 | 0.591 | 0.0627 | 2 / 6 | Moderate enrichment; the independent signature identified C1 as the highest-ranking component, indicating greater overlap among latent neuronal components in healthy tissue. |
| **E-MCI** | C2 | 0.758 | 1.12 × 10⁻⁵ | 1 / 6 | Strong enrichment. The independently selected marker set confirmed C2 as the best-matching inhibitory component. |
| **A-MCI** | C5 | 0.690 | 0.0018 | 2 / 6 | Significant enrichment. Although C2 achieved the highest AUC, C5 remained significantly enriched and therefore retained substantial inhibitory-neuron identity. |
| **AD** | C4 | 0.738 | 1.27 × 10⁻³ | 1 / 6 | Strong enrichment. Independent markers confirmed C4 as the component with the strongest inhibitory-neuron signature. |
